# Silica-coated magnetic nanoparticles activate microglia and induce neurotoxic d-serine secretion

**DOI:** 10.1186/s12989-021-00420-3

**Published:** 2021-08-12

**Authors:** Tae Hwan Shin, Da Yeon Lee, Balachandran Manavalan, Shaherin Basith, Yun-Cheol Na, Cheolho Yoon, Hyeon-Seong Lee, Man Jeong Paik, Gwang Lee

**Affiliations:** 1 Department of Physiology, Ajou University School of Medicine, 206 World cup-ro, 16499 Suwon, Republic of Korea; 2 Western Seoul Center, Korea Basic Science Institute, 150 Bugahyeon-ro, 03759 Seoul, Republic of Korea; 3 Ochang Center, Korea Basic Science Institute, 162 Yeongudanji-ro, 28119 Cheongju, Republic of Korea; 4 College of Pharmacy, Sunchon National University, 255 Jungang-ro, 57922 Suncheon, Republic of Korea; 5 Department of Molecular Science and Technology, Ajou University, 206 World cup-ro, 16499 Suwon, Republic of Korea

**Keywords:** Silica-coated magnetic nanoparticles, Nanotoxicity, Microglia, Excitotoxicity, Inclusion body

## Abstract

**Background:**

Nanoparticles have been studied for brain imaging, diagnosis, and drug delivery owing to their versatile properties due to their small sizes. However, there are growing concerns that nanoparticles may exert toxic effects in the brain. In this study, we assessed direct nanotoxicity on microglia, the resident macrophages of the central nervous system, and indirect toxicity on neuronal cells exerted by silica-coated magnetic nanoparticles containing rhodamine B isothiocyanate dye [MNPs@SiO_2_(RITC)].

**Methods:**

We investigated MNPs@SiO_2_(RITC)-induced biological changes in BV2 murine microglial cells *via* RNA-sequencing-based transcriptome analysis and gas chromatography-mass spectrometry-based intracellular and extracellular amino acid profiling. Morphological changes were analyzed by transmission electron microscopy. Indirect effects of MNPs@SiO_2_(RITC) on neuronal cells were assessed by Transwell-based coculture with MNPs@SiO_2_(RITC)-treated microglia. MNPs@SiO_2_(RITC)-induced biological changes in the mouse brain *in vivo* were examined by immunohistochemical analysis.

**Results:**

BV2 murine microglial cells were morphologically activated and the expression of Iba1, an activation marker protein, was increased after MNPs@SiO_2_(RITC) treatment. Transmission electron microscopy analysis revealed lysosomal accumulation of MNPs@SiO_2_(RITC) and the formation of vesicle-like structures in MNPs@SiO_2_(RITC)-treated BV2 cells. The expression of several genes related to metabolism and inflammation were altered in 100 µg/ml MNPs@SiO_2_(RITC)-treated microglia when compared with that in non-treated (control) and 10 µg/ml MNPs@SiO_2_(RITC)-treated microglia. Combined transcriptome and amino acid profiling analyses revealed that the transport of serine family amino acids, including glycine, cysteine, and serine, was enhanced. However, only serine was increased in the growth medium of activated microglia; especially, excitotoxic D-serine secretion from primary rat microglia was the most strongly enhanced. Activated primary microglia reduced intracellular ATP levels and proteasome activity in cocultured neuronal cells, especially in primary cortical neurons, *via *D-serine secretion. Moreover, ubiquitinated proteins accumulated and inclusion bodies were increased in primary dopaminergic and cortical neurons cocultured with activated primary microglia. *In vivo*, MNPs@SiO_2_(RITC), D-serine, and ubiquitin aggresomes were distributed in the MNPs@SiO_2_(RITC)-treated mouse brain.

**Conclusions:**

MNPs@SiO_2_(RITC)-induced activation of microglia triggers excitotoxicity in neurons *via *D-serine secretion, highlighting the importance of neurotoxicity mechanisms incurred by nanoparticle-induced microglial activation.

**Supplementary Information:**

The online version contains supplementary material available at 10.1186/s12989-021-00420-3.

## Background

Nanoparticles (NPs) for drug delivery, imaging, and diagnosis to the brain have been studied due to their ultra-small sizes. Some types of NPs, such as polymeric NPs [1], gold NPs [2], zinc oxide NPs [3], quantum dots [4], and silica-coated magnetic nanoparticles containing rhodamine B isothiocyanate dye [MNPs@SiO_2_(RITC)] [5] can cross the blood–brain barrier (BBB) through passive diffusion and endocytosis to access the brain. Notably, MNPs@SiO_2_(RITC) has been developed for cell labeling, separation, and magnetic resonance imaging contrast [6]. MNPs@SiO_2_(RITC) has a cobalt ferrite core and an RITC-containing silica shell; it can be detected using magnetic and fluorescence properties, thus enabling its broad application. Moreover, the silica shell is used for stability and biocompatibility owing to the highly toxic properties of cobalt ferrite [5, 7–9]. The toxicity of MNPs@SiO_2_(RITC) could not be detected by *in vitro* conventional methods, including fluorescence-activated cell sorting (FACS) cell death assay, cell viability assay, and chromosome aberration assay, in the HEK293 cell line and human bone marrow-derived mesenchymal stem cells [9–11]. Moreover, the pathological symptoms could not be detected by conventional methods, such as histopathological, growth, behavior, and serum biochemical analyses, in an *in vivo* mouse model [5, 11, 12]. However, the subtle toxicity of MNPs@SiO_2_(RITC) has recently been detected using transcriptomics, metabolomics, and mechanobiology [7–9, 13–15].

Accumulation of NPs in the brain is a serious concern owing to its toxic effects on CNS. Previous studies reported that NPs exposure and accumulation can induce neurodegeneration in brain by oxidative stress, deficits in motor and cognitive functions, neuronal deterioration, DNA damage by lipid peroxidation, neuroinflammatory response, microglia/astrocyte activation, and BBB damage/dysfunction [16]. In mice, MNPs@SiO_2_(RITC) crossed the BBB without disrupting it and accumulated in the brain for longer than 4 weeks, although toxicological symptoms were not detected using conventional methods [5].

Previous studies have found that protein fibrilization is observed in Alzheimer’s disease by NPs (cerium oxide particles, copolymer particles, carbon nanotubes, quantum dots) *in vitro* [17], nuclear protein aggregation by silica NPs is observed in a cellular model of Huntington’s disease, and formation of intracellular cytoplasmic inclusion bodies by MNPs@SiO_2_(RITC) in neuronal cells [8], may play a crucial role in neurodegenerative diseases. In addition, internalized metal-oxide nanoparticles in microglia-induced inflammation is a common factor in many neurodegenerative diseases [18]. However, studies related to neuro-nanotoxicity is very limited and its research area is still in infancy.

Microglia are resident macrophages which account for 5–20 % of the total brain cells. They are uniformly distributed [19] and activated by internalized NPs [20]. Upon activation by NPs, microglia secrete large amounts of ROS and nitric oxide (NO) and release various cytotoxic substances. The secreted substances can damage neurons and trigger neuronal death. For example, activated microglia impair neurons through *N*-methyl-D-aspartate (NMDA) receptor-mediated excitotoxicity by NMDA, polyamines, D-serine, and proteinogenic amino acid, such as glutamate and glycine [21, 22]. However, the toxicological mechanisms of activated microglia by NPs are not clearly understood.

Previously, we analyzed the direct effect of MNPs@SiO_2_(RITC) on the SH-SY5Y cell line, primary rat cortical neurons, and dopaminergic (DAergic) neurons [8]. Owing to their neuronal characteristics, SH-SY5Y neuroblastoma cells have been widely used in neuroscience research, including research on Parkinson’s disease, Alzheimer’s disease, neurotoxicity, ischemia, and amyotrophic lateral sclerosis; however, SH-SY5Y cells also exhibit genetic aberrations of cancerous origin [23]. MNPs@SiO_2_(RITC)-induced intracellular ROS generation, reduction in proteasome activity, and formation of inclusion bodies were evaluated in SH-SY5Y cells, cortical neurons, and DAergic neurons, and the changes were ranked in the following order according to their sensitivity to oxidative stress: DAergic neurons > cortical neurons > SH-SY5Y cells. In this study, we analyzed the indirect effect of MNPs@SiO_2_(RITC) on SH-SY5Y cells, cortical neurons, and DAergic neurons with respect to excitotoxicity using a co-culture with MNPs@SiO_2_(RITC)-treated microglial cells.

There are also few limitations in the evaluation of nanotoxicity using conventional methods due to its delicate complexities. Therefore, omics analyses have been used in the assessment of nanotoxicity through the quantification of gene expression, proteins, and metabolites for interpreting complex biological events and finding relationships between molecules and phenomena [11, 24]. Thus, integrated omics is indispensable to compensate for their related weaknesses and comprehensive analysis of nanotoxicity.

We investigated MNPs@SiO_2_(RITC)-induced biological changes in microglia by transcriptome analysis combined with amino acid profiling. Additionally, we examined the indirect effects of the MNPs@SiO_2_(RITC) on neuronal cells by co-culturing with MNPs@SiO_2_(RITC)-treated microglia. Finally, we validated MNPs@SiO_2_(RITC)-induced biological changes in the mouse brain *in vivo* through immunohistochemical analysis.

## Methods

### MNPs@SiO_2_(RITC) and silica NPs

MNPs@SiO_2_(RITC) composed of a ∼9-nm cobalt ferrite core (CoFe_2_O_3_) and an RITC-encompassed silica shell [6] were purchased from Biterials (Seoul, South Korea). The MNPs@SiO_2_(RITC) and SiO_2_ NPs were determined to be 50 nm in diameter using transmission electron microscopy (TEM) (Supplementary Fig. 1), and the MNPs@SiO_2_(RITC) had a zeta potential between − 40 and − 30 mV [6, 12]. X-ray diffraction (XRD) using a High-Power X-Ray Diffractometer (Ultima III, Rigaku, Japan) confirmed the structure of the MNPs@SiO_2_(RITC), which showed specific patterns of CoFe_2_O_4_: (220) at 30°, (311) at 36°, (400) at 44°, (511) at 57°, and (440) at 64°. The broad peak between 20° and 40° indicated amorphous silica beads (Supplementary Fig. 2). MNPs@SiO_2_(RITC) uptake was plateaued at 100 µg/ml MNPs@SiO_2_(RITC) treatment and cell viabilities of BV2 cell line and primary rat microglia were ~ 80 % reduced at 1.0 mg/ml MNPs@SiO_2_(RITC) treatment (data not shown). Thus, the MNPs@SiO_2_(RITC) treatment dose to microglia was determined as 10 to 100 µg/ml in this study.

### Primary cell isolation and cell culture

Primary cortical, DAergic neurons, and microglia were isolated from Sprague–Dawley rats (1 day pups). Briefly, rat brains were separated into cortex and midbrain and homogenized in Minimum Essential Medium (MEM, Gibco, NY, USA). The cell fractions were cultured in dishes containing MEM supplemented with 10 % fetal bovine serum (Gibco, NY, USA), 100 units/ml penicillin, and 0.1 mg/ml streptomycin (Gibco, NY, USA) in a humidified 5 % CO_2_ chamber at 37 °C. Primary rat microglia were isolated using the “shaking off” method [25]. The purity of the primary neuronal cells was verified using specific marker protein expression, i.e., anti-neuronal nuclei (NeuN) antibody (1:200, Abcam, USA) for cortical neuronal cells, anti-tyrosine hydroxylase (TH) antibody (1:200, Abcam, USA) for DAergic neuronal cells, anti-microtubule-associated protein 2 (MAP2) antibody (1:200, Novus Biologicals, USA) for neuronal cells, and anti-dopamine transporter (DAT) antibody (1:200, Novus Biologicals, USA) for DAergic neuronal cells. The purity of the primary rat microglia was verified by flow cytometry (BD Biosciences, San Jose, CA, USA) at the Three-Dimensional Immune System Imaging Core Facility of Ajou University after staining with a specific antibody (CD11b/c, integrin αM; clone OX-42, Santa Cruz, CA, USA) (Supplementary Fig. 3).

### Analysis of morphological changes in NPs treated microglia

To analyze NPs-induced microglia activation, BV2 and primary rat microglia cells were treated with 10 and 100 µg/ml of MNPs@SiO_2_(RITC) and SiO_2_ NPs or 10 and 100 ng/ml of LPS as activation control for 12 h. Images were acquired with an AxioVert 200 M fluorescence microscopy (Zeiss, Jena, Germany) at the Three-Dimensional Immune System Imaging Core Facility of Ajou University.

### Immunocytochemistry

After MNPs@SiO_2_(RITC) treatment or coculture on cover slips, cells were fixed in Cytofix buffer (BD Biosciences, CA, USA) at 4 °C for 30 min. PBS containing 2 % bovine serum albumin and 0.1 % Triton-X100 (Sigma-Aldrich, MO, USA) was used for blocking the cover slips. The cells were then incubated with anti-Iba1 goat polyclonal antibody (Novus biologicals, CO, USA), anti-D-serine rabbit polyclonal antibody (1:200, Abcam, Cambridge, UK), anti-vesicle-associated membrane protein 2 (VAMP2) mouse polyclonal antibody (1:200, GeneTex, CA, USA), anti- neuropeptide Y (NPY) mouse polyclonal antibody (1:200, GeneTex, CA, USA), anti-ubiquitin rabbit polyclonal antibody (1:200, Santa Cruz, CA, USA), and anti-alpha synuclein monoclonal antibody (1:200, BD Biosciences, CA, USA) diluted in blocking buffer at 4 °C for 12 h. After three washes with PBS containing 0.1 % Triton X-100, they were incubated with Alexa Fluor 488-, 547-, and 647-conjugated secondary antibodies (1:200, Invitrogen, CA, USA) at room temperature (RT) for 1 h. The labeled cells were washed thrice with PBS containing 0.1 % Triton X-100 and incubated with PBS containing 10 µg/ml Hoechst 33,342 at RT for 10 min. After three washes with PBS, the cover slips were mounted onto slides using Prolong Gold Antifade mounting medium (Molecular Probes, OR, USA). Fluorescence images were taken using a slide scanner (Axioscan Z1, Carl Zeiss Microscopy GmbH, Jena, Germany) or by high-resolution confocal microscopy (Nikon A1, Nikon, Tokyo, Japan) at the Three-Dimensional Immune System Imaging Core Facility of Ajou University. To quantify inclusion bodies, total cells were counted using ImageJ (NIH Image, Bethesda, MD) and the frequency of aggresome-containing cells was calculated.

### TEM

To analyze ultrastructural changes, Karnovsky’s fixative (Sigma-Aldrich, MO, USA) was used for MNPs@SiO_2_(RITC)-treated BV2 cells fixation at 4 °C for 12 h. After fixation, samples were washed with 0.1 M cacodylate buffer (pH 7.4). Cacodylate buffer (0.1 M) with osmium tetroxide [1 % (v/v), Polysciences, PA, USA] was used for post-fixation at RT for 2 h. The samples were dehydrated with graded ethanol solutions (50–100 %), infiltrated with propylene oxide, and embedded in Epon (Polysciences, PA, USA). The samples were incubated at 35 °C for 6 h, 45 °C for 12 h, and 60 °C for 24 h. The blocks were sectioned using an ultramicrotome (Reichert-Jung, Bayreuth, Germany). The sections were double-stained with 6 % uranyl acetate (Electron Microscopy Sciences, PA, USA) for 20 min and lead citric acid for 10 min (Thermo Fisher Scientific, CA, USA) for contrast. Images were obtained using a SIGMA500 transmission electron microscope (Carl Zeiss Microscopy GmbH, Jena, Germany) at the Three-Dimensional Immune System Imaging Core Facility of Ajou University. Particle number, vesicle size, mitochondria number, and size were analyzed using the Zen blue 2.3 (Carl Zeiss Microscopy GmbH, Jena, Germany) image analysis module.

### Inductively Coupled Plasma Mass Spectrometry (ICP-MS)

MNPs@SiO_2_(RITC) in BV2 were quantified by ICP-MS (7700, Agilent, Japan) as reported [6]. Briefly, BV2 cells were incubated with 0, 10, or 100 µg/ml MNPs@SiO_2_(RITC) for 12 h, and 5 × 10^6^ cells were collected. The treated cells and MNPs@SiO_2_(RITC) were digested and completely dissolved in concentrated aqua regia and hydrogen fluoride (HF). CoFe_2_O_4_, which is the core of the MNPs@SiO_2_(RITC), contains approximately 10^− 18^ mmol cobalt ions. Thus, the number of MNPs@SiO_2_(RITC) in each BV2 cell was calculated.

### RNA-seq

To analyze transcriptome of control BV2 cells and 10 or 100 µg/ml MNPs@SiO_2_(RITC) treated BV2 cells, total RNA was extracted using a TruSeq Stranded Total RNA Library Prep Kit (Illumina, CA, USA). An Agilent RNA 6000 Nano Kit (Agilent Technologies, Waldbronn, Germany) was used for analyzing RNA quality. mRNA was enriched using Magnetic beads conjugated with oligo(dT). Double-stranded cDNA was synthesized with purified mRNA after fragmentation. Sequencing adapters using a TruSeq RNA Sample Prep Kit (Illumina, CA, USA) was used for modification, including end-repair and poly(A) addition and connection, of the cDNA. The samples were isolated using Blue Pippin (Sage Science, MA, USA). cDNA library size and quality were determined using an Agilent High Sensitivity DNA Kit (Agilent Technologies, Waldbronn, Germany). The libraries were sequenced using an Illumina HiSeq2500 sequencer (Illumina, CA, USA).

### Differentially Expressed Gene (DEG) analysis

Low-quality reads (reads containing more than 10 % skipped bases, reads containing more than 40 % of bases with quality scores < 20, and reads with an average quality score < 20) were filtered from the RNA-seq data using in-house scripts. Filtered reads were identified using the aligner TopHat [26]. Expression levels were calculated using Cufflinks v2.1.1 [26] and Ensembl, release 77. Multi-read-correction and fragbias- correct were used for increasing the measurement accuracy. DEGs were singled out using Cuffdiff with default parameter settings, based on *p* < 0.05.

### Gene Ontology (GO) and pathway analyses

The GO database classifies genes into functional categories and can be used to predict gene function based on Mouse Genome Informatics (MGI) data [27]. Biological changes, related with canonical pathways and functions, were analyzed using an ingenuity pathway (IPA) web-based software (Qiagen, CA, USA). A 1.5-fold change in gene expression and a 1.2-fold change in amino acid levels were used as cut-offs.

### Quantitative reverse transcription PCR (RT-qPCR)

To quantify gene expression levels of MNPs@SiO_2_(RITC) BV2 cells, RNA extraction and cDNA construction for RT-qPCR were performed as previously study [8]. The cells were lysed using RNAzol B (Tel-Test, TX, USA). Chloroform (Sigma-Aldrich, MO, USA) and isopropyl alcohol (Sigma-Aldrich, MO, USA) were used for total RNA precipitation. Pellets were washed with 70 % ethanol and the samples were dissolved in RNase-free water. The purities of samples were measured by 260 nm/230 nm and 260 nm/280 nm absorbance ratio using spectrophotometry (Eppendorf, Hamburg, Germany). cDNA was synthesized using the iScript Advanced cDNA Synthesis Kit (Bio-Rad, Hercules, CA, USA). The thermal cycle was as follows: 46 °C for 20 min followed by 95 °C for 1 min.

Expression levels of genes were quantified by qPCR using SsoAdvanced™ Universal SYBR® Green Supermix (Bio-Rad) and gene-specific primers (Supplementary Tables 1 and 2﻿) on a Rotor Gene-Q system (Qiagen, CA, USA). Thermal cycles were as follows: 95 °C for 5 min, followed by 50 cycles of 95 °C for 5 s and 60 °C for 30 s. Target gene expressions were calculated with melting curves by the 2^−ΔΔCt^ method.

### Gas chromatography (GC)-MS

The amino acid content in the MNPs@SiO_2_(RITC)-treated BV2 cells and media was analyzed using GC-MS as reported previously [9]. Norvaline is used as internal standard. GC-MS was conducted in both the scan and selected-ion monitoring (SIM) modes on a 6890 N gas chromatograph (Agilent Technologies, Santa Clara, CA, USA) interfaced with a 5975B mass-selective detector (70 eV, electron impact ionization mode; Agilent Technologies).

### Determination of D-serine secreted from microglia

D-Serine concentrations in the medium were determined using a D-serine colorimetric assay (Cosmo Bio, Tokyo, Japan) per the manufacturer’s instructions. Briefly, medium of the MNPs@SiO_2_(RITC) or lipopolysaccharide (LPS)-treated BV2 cells and primary rat microglia were mixed with NADH and D-serine dehydratase from *Saccharomyces cerevisiae* (DsdSC), D-serine to pyruvate converting enzyme, at 37 °C for 45 min. D-serine samples incubated with nicotinamide adenine dinucleotide (NADH), but without DsdSC were used as blank. The absorbance at 340 nm was measured using a microplate reader (Molecular Devices, CA, USA). Lactate dehydrogenase (LDH) was added to the mixed samples and incubated at 37 °C for 45 min. The pyruvate to lactate conversion by LDH leaded to reduction in optical density at 340 nm by NADH oxidation. The D-serine concentration was quantified by calculating the reduction absorbance at 340 nm and a D-serine standard curve.

### Liquid Chromatography (LC)-MS

L- and D-Serine were purchased from Tokyo Chemical Industry (Tokyo, Japan). HPLC-grade acetonitrile, ethanol, and trifluoroacetic acid were obtained from Thermo Fisher Scientific (CA, USA). Stock solutions of L- and D-serine were prepared at 1 mg/mL in water. A standard stock solution of amino acids was diluted to 0.01–100 µg/ml for calibration. Calibration curves were plotted with seven points using the peak areas obtained for D- and L-serine versus the concentration by linear regression. The samples were centrifuged at 12,000 ⋅ *g* for 15 min. The supernatant was filtered through a 3-kDa filter (Millipore, Darmstadt, Germany) by centrifugation at 14,000 ⋅ *g* at 4 °C for 30 min. The filtered aliquot was used for analysis.

Chiral DL-serine separations were performed using an Agilent 1290 infinitely LC (Agilent Technologies, Walbronn, Germany) using a CROWNPAK CR-I(+) (3.0 × 150 mm, 5 μm, Chiral Technologies) at 30 °C. Mobile phase comprising acetonitrile: ethanol: water: trifluoroacetic acid (80: 15: 5: 0.5) was employed at a flow rate of 0.4 ml/min. Two microliters of sample were injected into the column using a thermostated HiP-ALS autosampler. The HPLC system was interfaced with an Agilent 6495 Q-TOF (Agilent Technologies, Walbronn, Germany) mass spectrometer. The source conditions were set to a Vcap of 3.5 kV in positive ESI mode with a sheath gas temperature of 200 °C and a sheath gas flow rate of 11 ml/min. The DL-serine was detected by multiple reaction monitoring. A product ion at m/z 60 fragmented by a collision energy of 10 V from the precursor ion at m/z 106 was monitored for 200 ms.

### Evaluation of neuronal cell viability in a microglia coculture system

The coculture system was set up as reported previously [28]. Briefly, neuronal cells or SH-SY5Y cells were cultured on the bottom side of a Costar Transwell plate (0.4-µm pore size; Corning, NY, USA) in a humidified 5 % CO_2_ incubator at 37 °C for 24 h. Microglia in the culture inserts were treated with MNPs@SiO_2_(RITC) or LPS for 12 h. Then, the inserts were placed in the plate and incubated for another 12 h. To measure the viability of the neuronal cells, the upper inserts were removed, and 3-(4,5-dimethylthiazol-2-yl)-5-(3-carboxymethoxyphenyl)-2-(4-sulfophenyl)-2 H-tetrazolium solution (CellTiter 96® AQueous One; Promega, WI, USA) was added to each well containing the microglia. The assay plate was incubated at 37 °C for 1 h. The amount of soluble formazan produced *via* cellular reduction was measured using a plate reader (Molecular Devices, CA, USA) at 490 nm. Values were normalized to the corresponding total protein. Images of cell morphology and density were acquired before the cell viability assay under an Axiovert 200 M fluorescence microscope (Carl Zeiss Microscopy GmbH, Jena, Germany) at the Three-Dimensional Immune System Imaging Core Facility of Ajou University. The excitation wavelength for the MNPs@SiO_2_(RITC) was 530 nm.

### Measurement of the ATP concentration

The ATP concentration of the microglia cocultured neurons and D-serine treated neurons were determined using an ATP quantification kit (Promega, WI, USA) per the manufacturer’s protocol. Briefly, the neuronal cells were detached from plate and washed with PBS. Intracellular ATP was extracted with a 1.0 % trichloroacetic acid (Sigma Aldrich, MO, USA) at RT for 30 min. The extractant were mixed with a luciferin reagent/buffer mixture and then split in white 384-well plates. After 10 min of incubation at 25 °C, the luminescence in each well was measured using a luminometer (LMaxII^384^; Molecular Devices, CA, USA).

### Proteasome activity assay

Proteasome activity was measured using a Proteasome-Glo™ Chymotrypsin-Like Cell-Based Assay (Promega, WI, USA) per the manufacturer’s protocol. Briefly, microglia cocultured neurons and D-serine treated neurons were washed three times with PBS, replenished with 50 µl of serum-free medium, and left at RT. Proteasome-Glo™ cell-based buffer was mixed with luciferin detection agent and appropriate Proteasome-Glo™ substrate and incubated at RT for 30 min. Fifty-microliter aliquots of this mixture were then added to the medium in each well and incubated at RT for 10 to 15 min. Next, 90 µl of the medium containing a proteasome assay solution was transferred to corresponding wells in a white plate. Proteasome activity was determined by measuring the chymotrypsin-like activity of cellular proteasomes using the luminogenic substrate Suc-Leu-Leu-Val-Tyr-aminoluciferin in a luminometer (LMaxII^384^; Molecular Devices, CA, USA) per the manufacturer’s instructions.

### Evaluation of D-serine distribution and inclusion body formation *in vivo*

All animal experimental protocols were approved by the Laboratory Animal Research Center of Ajou University Medical Center (approval no. 2020-0033) and complied with the institutional ethical use protocols (NIH Guide for Care and Use of Laboratory Animals). Male ICR mice (8 weeks old, Orientbio, Seongnam, Korea) were maintained under 12-h light/dark cycles with free access to food and water. Four mice per group were used in this study. The biodistribution and toxicological effects of the MNPs@SiO_2_(RITC) at 25, 50, and 100 mg/kg were previously reported [5]. The study showed the broad-range tissue distribution of the MNPs@SiO_2_(RITC) and the brain localization of the MNPs@SiO_2_(RITC) without blood–brain barrier disruption. The particles did not induce significant toxicological symptoms in terms of growth, behaviors, biochemical changes in serum, and histopathology, even at 100 mg/kg. However, based on our *in vitro* findings, we postulated that there would be subtle toxicity in the brain at 100 mg/kg, which was the maximum concentration evaluated in the previous study. Thus, MNPs@SiO_2_(RITC) were injected intraperitoneally with sterile saline at 100 mg/kg per mouse. Control mice were injected with sterile saline only. The endpoint was determined at 5 days, based on a previous biodistribution study [5]. Five days after the injection, the mice were anaesthetized with urethane (1.2–1.5 g/kg, intraperitoneally). The hearts were rapidly exposed, and the mice were transcardially perfused with paraformaldehyde (PFA, Sigma-Aldrich, MO, USA). The brains of the PFA-perfused mice were removed and placed in PFA for 24 h. The brains were cryoprotected by immersion in 30 % sucrose and then sectioned at 5 μm and stored at − 80 °C until analysis.

Frozen sections were incubated in blocking solution containing 1 % bovine serum albumin (Sigma-Aldrich, MO, USA) and 10 % donkey serum (Sigma-Aldrich, MO, USA) in PBS at RT for 2 h. The sections were stained with anti-D-serine rabbit polyclonal antibody (1:200, Abcam, Cambridge, UK), anti-Iba1 goat polyclonal antibody (1:100, Novus Biologicals, CO, USA), and anti-ubiquitin rabbit polyclonal antibody (1:200, Santa Cruz, CA, USA) diluted in 1 % donkey serum in PBS with 0.4 % Triton X-100 at 4 °C overnight. The sections were rinsed and washed (three times for 10 min) in PBS with 0.4 % Triton X-100 and incubated with Alexa Fluor 488- or 647-conjugated secondary donkey antibodies (Invitrogen, CA, USA) at 1:100 dilution in 1 % donkey serum in PBS with 0.4 % Triton X-100 at RT for 2 h. After the wash steps, the sections were mounted with Vectashield mounting medium containing DAPI (Vector Laboratories, CA, USA), and coverslips were applied. Immunostained sections were scanned using a slide scanner (Axio Scan Z1, Carl Zeiss Microscopy GmbH, Jena, Germany), and regions of interest were viewed under a 40× objective lens on an A1R HD25 confocal microscope (Nikon, Tokyo, Japan) at the Three-Dimensional Immune System Imaging Core Facility of Ajou University. Three-dimensional reconstructions of branch structures were acquired and quantified for number and length, using Imaris 9.2 software (Bitplane, Zurich, Switzerland) at the Three-Dimensional Immune System Imaging Core Facility of Ajou University.

### Statistical analysis

Data were analyzed by analysis of variance (ANOVA) with Bonferroni’s multiple-comparison tests, using IBM-SPSS software (IBM, NY, USA). Differences were considered significant at *p* < 0.05.

## Results

### Phenotype changes and NP uptake efficiency of MNPs@SiO_2_(RITC)-treated microglia

**Fig. 1:**
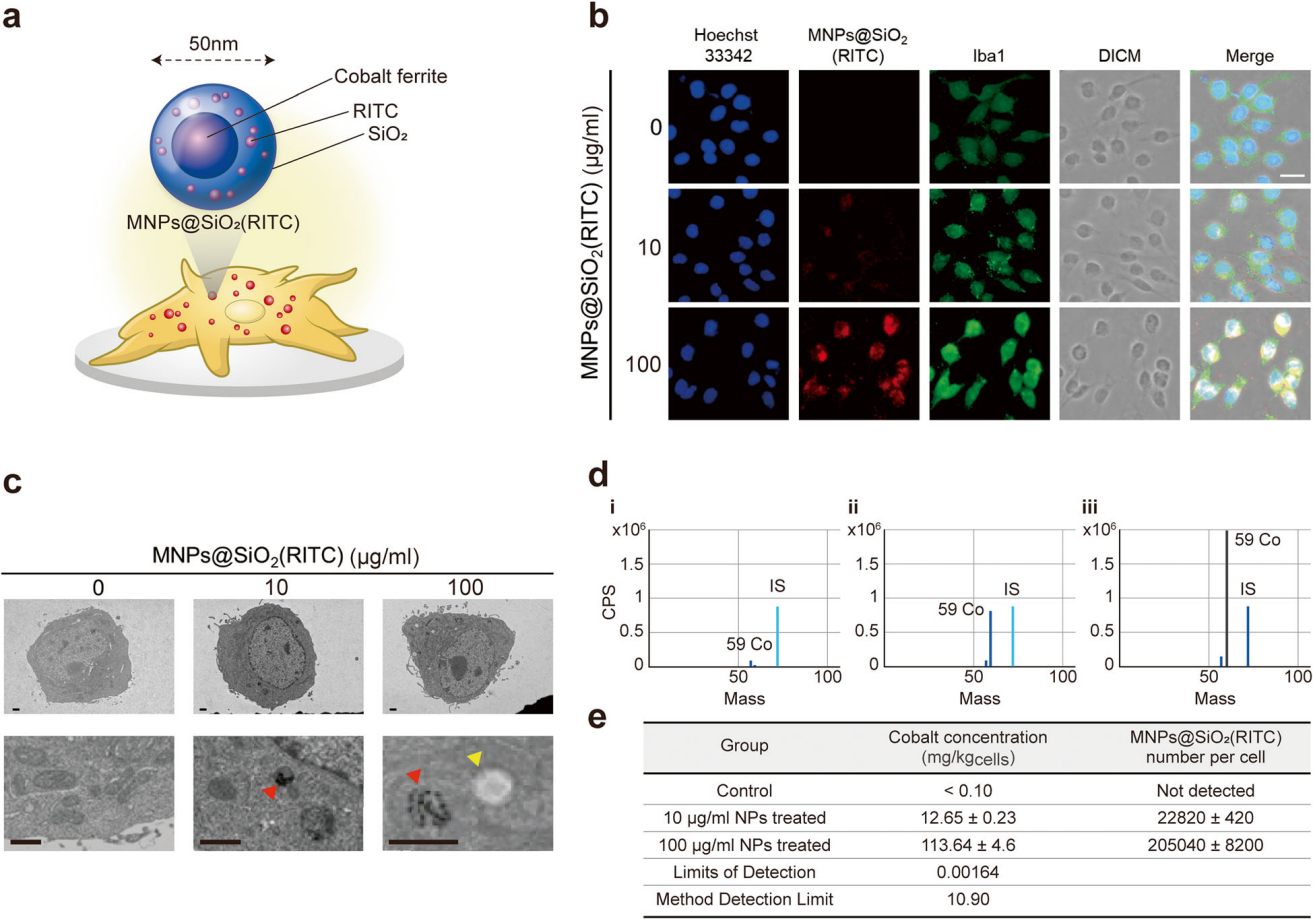
TEM analysis of MNPs@SiO_2_(RITC)-treated BV2 cells.** a** Schematic representation of the MNPs@SiO_2_(RITC) composition. **b** Immunostaining image of Iba1 (green), MNPs@SiO_2_(RITC) (red), and the nucleus (blue). Scale bar = 20 μm. **c** Representative TEM images of MNPs@SiO_2_(RITC)-treated BV2 cells. Magnified images are presented in the bottom panels. The red arrow head indicates MNPs@SiO_2_(RITC) in the cell, and the yellow arrow head indicates a granular structure. Scale bar = 1 μm. **d** ICP-MS peak of the cobalt ion in the control and MNPs@SiO_2_(RITC)-treated microglia. IS = internal standard. **e** Quantification of the cobalt concentration and MNPs@SiO_2_(RITC) particles in each MNPs@SiO_2_(RITC)-treated cell

To evaluate the effect of MNPs@SiO_2_(RITC) on microglia, we first analyzed morphological changes in MNPs@SiO_2_(RITC)-treated BV2 immortalized murine microglial cells and primary rat microglia. The cells were treated for 12 h with 10 or 100 µg/ml MNPs@SiO_2_(RITC) (Fig. 1a) or with 0 (non-treated control), 10, or 100 ng/ml of LPS as a positive control for morphological activation. Morphological activation (i.e., swollen and round cells) was observed in MNPs@SiO_2_(RITC)- and LPS-treated microglia (Supplementary Fig. 4). Compared to non-treated control cells, BV2 cells and primary rat microglia showed dose-dependent morphological changes following MNPs@SiO_2_(RITC) or LPS treatment, but there was no significant difference in cell density.

We previously suggested that the biological changes in HEK293 cells were induced by the shell (silica) rather than the cobalt ferrite core of MNPs@SiO_2_(RITC) [7, 8, 11, 13–15]. Moreover, our previous studies revealed that these biological changes, which included cell viability, intracellular ROS generation, and proteasome activity, were similar between MNPs@SiO_2_(RITC)- and silica NP-treated HEK293 cells. In this study, we also analyzed morphological changes in silica NP-treated microglia, which exhibited similar trends between MNPs@SiO_2_(RITC)- and silica NP-treated microglia (Supplementary Fig. 5).

Iba1 is a microglial marker whose expression is elevated upon microglial activation [29]. MNPs@SiO_2_(RITC)-treated BV2 cells were stained for Iba1 after 10 or 100 µg/ml MNPs@SiO_2_(RITC) treatment for 12 h (Fig. 1b). Iba1-staining fluorescence was increased in a MNPs@SiO_2_(RITC) dose-dependent manner. Thus, MNPs@SiO_2_(RITC) treatment induces morphological and biological activation in microglia.

To evaluate detailed changes at the organelle level, the MNPs@SiO_2_(RITC)-treated BV2 cell ultrastructure was visualized using TEM. MNPs@SiO_2_(RITC) were clearly visible as black dots throughout the cytoplasm, and the mitochondria showed the most pronounced changes in size and number among the organelles (Fig. 1c). MNPs@SiO_2_(RITC) were not distributed in the nucleus. The lysosomes, containing MNPs@SiO_2_(RITC), were approximately 800 nm in diameter. The number of granular structures was increased by ~ 3-fold in 100 µg/ml MNPs@SiO_2_(RITC)-treated BV2 cells compared to 10 µg/ml MNPs@SiO_2_(RITC)-treated and control cells.

In this study, BV2 cells were treated with 10 or 100 µg/ml MNPs@SiO_2_(RITC), and the uptake efficiency was determined by ICP-MS (Fig. 1d, e). The efficiency was approximately 2.3 × 10^4^ particles/cell at 10 µg/ml MNPs@SiO_2_(RITC) and 2.0 × 10^5^ particles/cell at 100 µg/ml MNPs@SiO_2_(RITC).

### Transcriptome analysis of MNPs@SiO_2_(RITC)-treated BV2 cells

To evaluate the biological changes in MNPs@SiO_2_(RITC)-treated BV2 cells, transcriptome changes after treatment with 10 or 100 µg/ml MNPs@SiO_2_(RITC) were investigated using RNA-sEq. Changes in gene expression were more pronounced at 100 µg/ml than at 10 µg/ml. DEGs in 100 µg/ml MNPs@SiO_2_(RITC)- vs. 10 µg/ml MNPs@SiO_2_(RITC)-treated cells were subjected to GO analysis (Supplementary Fig. S6a); 29 DEGs (51.5 % of the total DEGs) were engaged in metabolism, and 18 DEGs (23.9 %) were related to signal transduction, especially inflammation (Supplementary Fig. S6b). A gene co-expression network based on the RNA-seq data was constructed using IPA. The genes in the network are presented in relation to their function in metabolism and inflammation (Supplementary Fig. S6c, Supplementary Tables 3, and Supplementary Fig. 7). The expression levels of the genes in the network were quantified using RT-qPCR (Supplementary Fig. S6d). In particular, the expression of chemokine (C-C motif) receptor 1 (*Ccr1*), colony-stimulating factor 3 (*Csf3*), and chemokine (C-X-C motif) ligand 3 (*Cxcl3*) was significantly upregulated and that of formyl peptide receptor 1 (*Fpr1*) and serine (or cysteine) peptidase inhibitor, clade F, member 1 (*Serpinf1*) was significantly downregulated in 100 µg/ml MNPs@SiO_2_(RITC)-treated cells compared to non-treated control cells.

### Disturbances in intracellular amino acids in MNPs@SiO_2_(RITC)-treated microglia

**Fig. 2:**
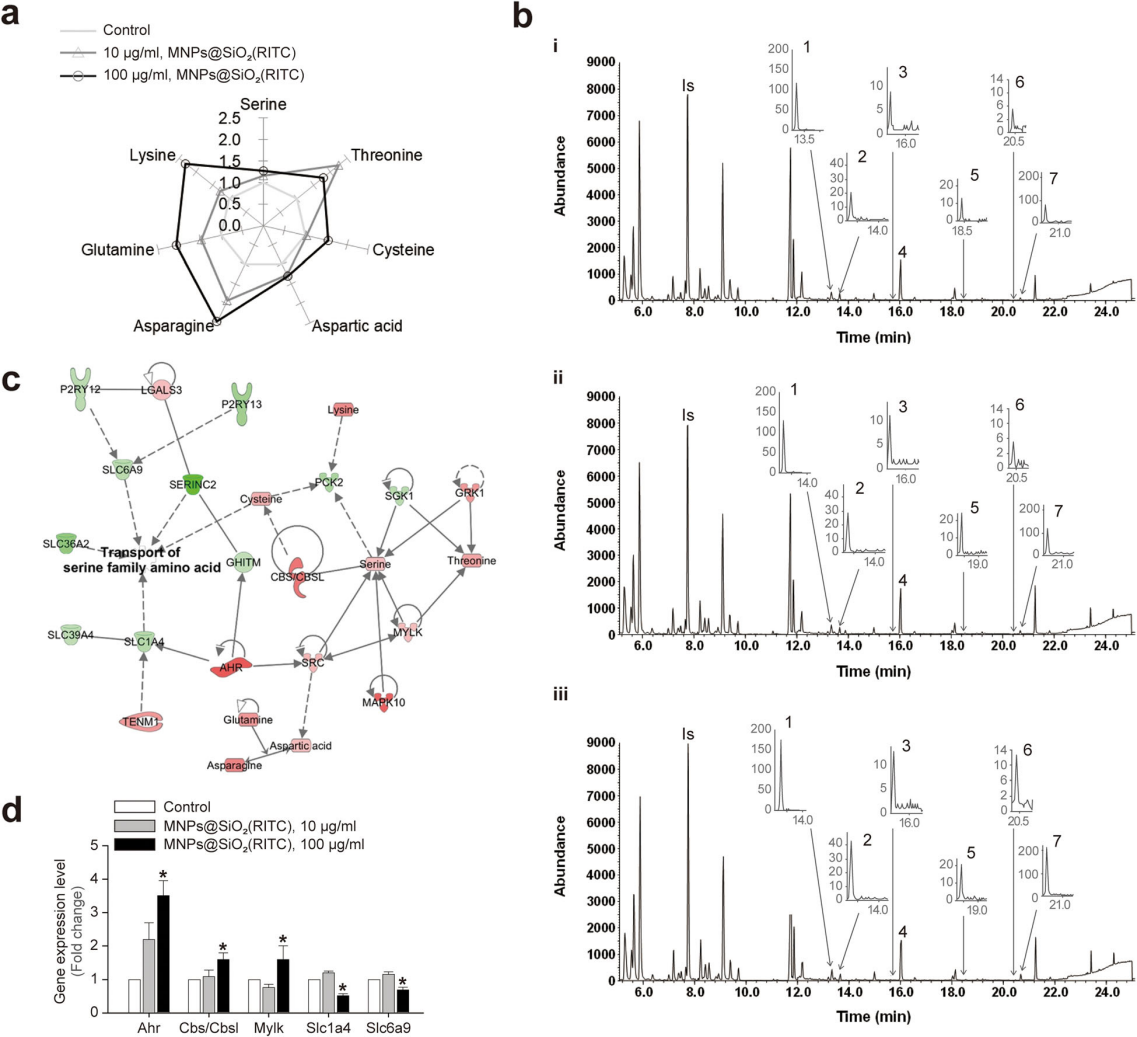
Combined RNA-seq and intracellular amino acid profiling analyses of BV2 cells treated with MNPs@SiO_2_(RITC) for 12 h.** a** Star plot showing altered levels of 7 out of 19 amino acids evaluated in BV2 cells after MNPs@SiO_2_(RITC) treatment. Target levels were normalized to the corresponding values in the non-treated control group. **b** SIM chromatograms of the 7 amino acids in (**i**) control, (**ii**) 10 µg/ml, and (**iii**) 100 µg/ml MNPs@SiO_2_(RITC)-treated BV2 cells. **c** Transcriptome and amino acid network for 100 µg/ml MNPs@SiO_2_(RITC)-treated BV2 cells constructed IPA. Red and green areas indicate the up- and downregulated genes, respectively. As cut-offs, fold changes > 1.5 or <–1.5 for genes and of > 1.2 or <–1.2 for amino acids were used. **d** RT-qPCR analysis of gene expression in each group, using *GAPDH* as an internal control. Data represent the mean ± standard deviation of three independent experiments. *p < 0.05 vs. non-treated control

We profiled 19 cellular amino acids in 10 or 100 µg/ml MNPs@SiO_2_(RITC)-treated BV2 cells by ethoxycarbonyl (EOC)/*tert*-butyldimethylsilyl (TBDMS) derivatization and GC-MS (Supplementary Table 4). A star plot revealed increased levels of 7 of the 19 amino acids, including serine, threonine, cysteine, aspartic acid, asparagine, glutamine, and lysine (Fig. 2a). Representative SIM chromatograms showing the changes in the amounts of these seven amino acids in control, 10 µg/ml MNPs@SiO_2_(RITC)-treated, and 100 µg/ml MNPs@SiO_2_(RITC)-treated cells are shown in Fig. 2b, i–iii.

Amino acid profiles and a gene co-expression network were constructed using IPA. The network revealed that changes in the expression of genes related to transport function were highly associated with changes in serine family amino acids, including glycine, cysteine, and serine, in 100 and 10 µg/ml MNPs@SiO_2_(RITC)-treated cells compared to non-treated cells (Fig. 2c, Supplementary Tables 5, and Supplementary Fig. 8). The expression levels of aryl-hydrocarbon receptor (*Ahr*), cystathionine beta-synthase (*Cbs/Cbsl*), and 130-kDa myosin light chain kinase (*Mylk*) were significantly increased, whereas those of solute carrier family 1 (glutamate/neutral amino acid transporter), member 4 (*Slc1a4*) and solute carrier family 6 (neurotransmitter transporter, glycine), and member 9 (*Slc6a9*) were significantly decreased in 100 µg/ml MNPs@SiO_2_(RITC)-treated cells compared to control cells as indicated by RT-qPCR (Fig. 2d).

### D-Serine secretion in MNPs@SiO_2_(RITC)-treated microglia *in vitro*

**Fig. 3:**
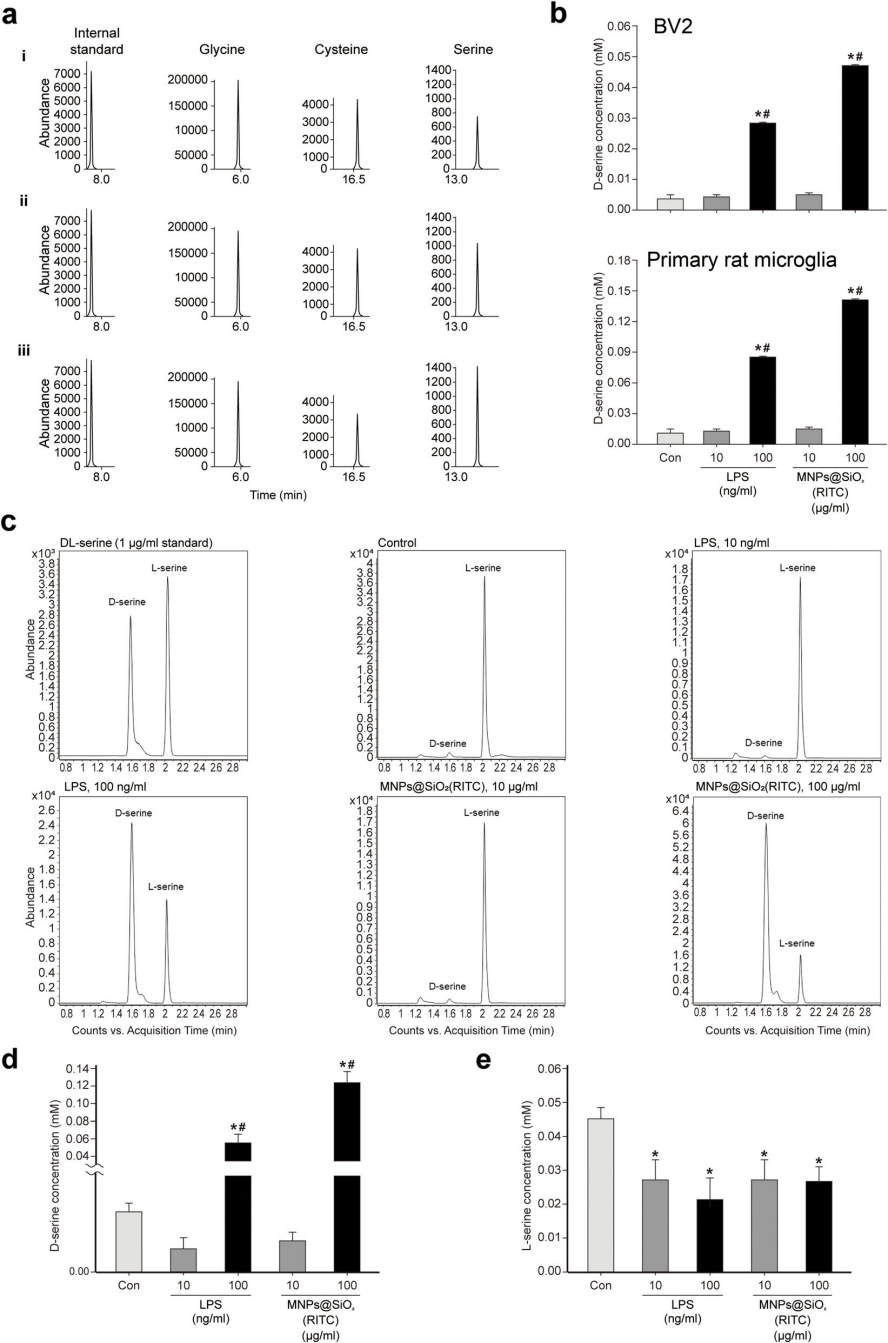
Analysis of the changes in serine family amino acids and quantification of L-and D-serine secretion by BV2 cells treated with MNPs@SiO_2_(RITC). **a** SIM chromatograms of glycine, cysteine, and serine in (**i**) control (**ii**) 10 µg/ml, and (**iii**) 100 µg/ml MNPs@SiO_2_(RITC)-treated microglia. The internal standard was norvaline. **b** Quantitative analysis of D-serine in the medium of MNPs@SiO_2_(RITC)-treated microglial cells (top panel: BV2 cells, bottom panel: primary rat microglia) using an enzyme-based system. Data were normalized to the control and represent the mean ± SD of three independent experiments. *p < 0.05 vs. non-treated control. **c** Multiple reaction monitoring peaks of L- and D-serine in the control, 10 µg/ml, and 100 µg/ml MNPs@SiO_2_(RITC)- and 10 and 100 ng/ml LPS-treated primary rat microglia. Quantification and statistical analysis of L- **d** and D-serine **e** in control, 10 µg/ml, and 100 µg/ml MNPs@SiO_2_(RITC)- and 10 and 100 ng/ml LPS-treated primary rat microglia. *p < 0.05 vs. non-treated control. ^#^*p* < 0.05 vs. 10 µg/ml MNPs@SiO_2_(RITC)-treated cells

To validate the above findings, we analyzed the extracellular secretion of the above 19 amino acids, including serine family amino acids, in the medium of MNPs@SiO_2_(RITC)-treated BV2 cells using EOC/TBDMS derivatization and GC-MS (Supplementary Table 6). The increase in serine in the medium was significantly higher than the increases in glycine and cysteine. The representative SIM chromatograms corroborated that serine was more strongly increased in the medium than other serine family amino acids (Fig. 3a). The peak area ratios of serine to the internal standard were approximately 0.10 (control; Fig. 3a, i), 0.12 (10 µg/ml treatment; Fig. 3a, ii), and 0.16 (100 µg/ml treatment; Fig. 3a, iii).

The GC-MS platform used in this study could not distinguish enantiomers of serine; therefore, we measured D-serine in the media of MNPs@SiO_2_(RITC)-treated BV2 cells and primary rat microglia using a D-serine-specific enzyme-based method. D-Serine secretion was increased after LPS and MNPs@SiO_2_(RITC) treatment in BV2 cells and primary microglia (Fig. 3b). Specifically, the increase in D-serine secretion was the most pronounced in 100 µg/ml MNPs@SiO_2_(RITC)-treated primary microglia. To validate the aforementioned results of the indirect method, we reanalyzed D-serine by liquid chromatography mass spectrometry, which is a direct detection method for pure D-serine level, using an enantio-separation column. L- and D-serine were clearly separated, and the extracellular levels of D-serine were increased in 10 ng/ml LPS- and 10 µg/ml MNPs@SiO_2_(RITC)-treated primary rat microglia; the changed levels exhibited a similar trend and degree (difference within ± 10 %) in both the indirect and direct methods (Fig. 3c, d). L-Serine levels were rather decreased in LPS- and MNPs@SiO_2_(RITC)-treated cells compared to those in control cells (Fig. 3e).

### ATP and proteasome activity are reduced in neuronal cells cocultured with microglia

**Fig. 4:**
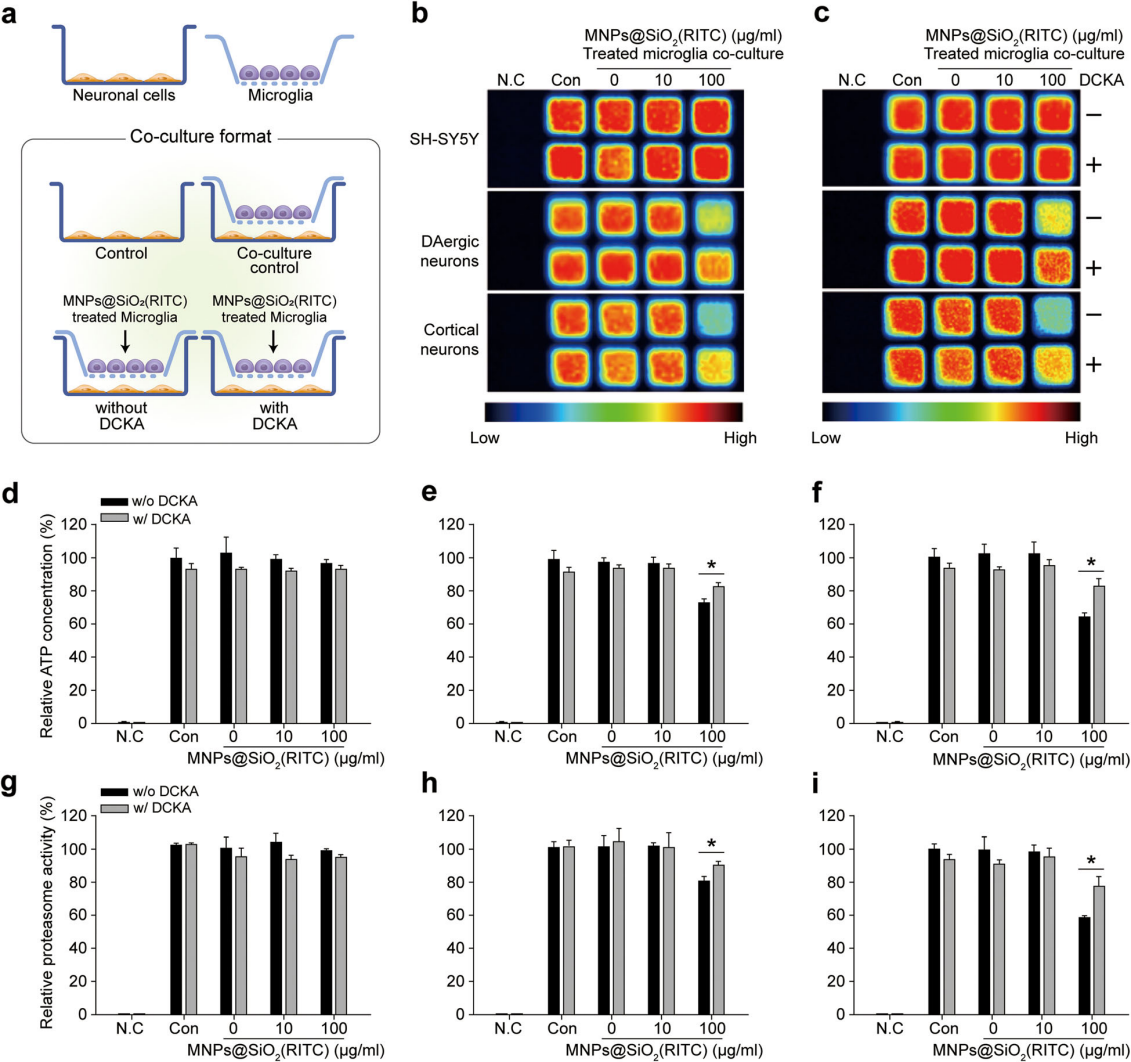
Evaluation of the intracellular ATP level and proteasomal activity in MNPs@SiO_2_(RITC)-treated microglia-cocultured neurons.** a** Schematic representation of the primary rat microglia and neuronal cell coculture system. Luminescence images of the ATP level **b** and proteasomal activity **c**. N.C.: non-treated control control; Con: neuronal cells only; DCKA: 5,7-dichlorokynurenic acid. Plus and minus signs indicate with or without DCKA treatment. Statistical analysis of ATP levels in **d** SH-SY5Y, **e** DAergic, and **f** cortical neurons. Statistical analysis of proteasomal activity in **g** SH-SY5Y, **h** DAergic, and **i** cortical neurons. *p < 0.05 vs. without DCKA. The experiments were repeated three times independently, and a representative result is shown

To evaluate the effect of activated microglia and secreted D-serine on neuronal cells, we used a Transwell coculture system (Fig. 4a). Microglia, activated with 10 or 100 µg/ml MNPs@SiO_2_(RITC) or 10 or 100 ng/ml LPS for 12 h, were cocultured with SH-SY5Y, cortical, and DAergic neurons. After 12 h, the morphology and density of the neuronal cells were observed by microscopy, and cell viability was analyzed (Supplementary Fig. 9). The viability of cortical neurons was decreased, and aggresome-like structures were detected after coculture with 100 µg/ml MNPs@SiO_2_(RITC)-activated microglia. However, cell viability and density were not affected in MNPs@SiO_2_(RITC)-activated microglia-cocultured SH-SY5Y cells and DAergic neurons.

To evaluate the effect of D-serine secreted from the activated microglia on neuronal cells, we first focused on the mitochondria, because calcium ions from the excitotoxic cascade mainly impair calcium channel-containing organelles such as the mitochondria and the endoplasmic reticulum [30], and the apoptosis cascade is highly associated with the mitochondria [31]. The mitochondrial membrane potential (Δψ_m_) was analyzed in neuronal cells cocultured with MNPs@SiO_2_(RITC)-activated microglia (Supplementary Fig. 10). Populations with disrupted Δψ_m_ were increased in the neuronal cells cocultured with 100 µg/ml MNPs@SiO_2_(RITC)-activated microglia, and the most pronounced decrements (~ 30 %) were observed in cortical neuronal cells. The decrease in Δψ_m_ was alleviated in the presence of an antagonist of the D-serine binding site of the NMDA receptor, 5,7-dichlorokynurenic acid (DCKA).

Decreases in Δψ_m_ are highly correlated with decreases in mitochondrial function, specifically ATP production [32]. Intracellular ATP was measured in neuronal cells cocultured with MNPs@SiO_2_(RITC)-activated microglia. Intracellular ATP levels were reduced in neuronal cells cocultured with 100 µg/ml MNPs@SiO_2_(RITC)-activated microglia, and the most pronounced decrease was observed in cortical neuronal cells (Fig. 4b). The decrease in ATP was also alleviated in the presence of DCKA.

Proteasome activity is highly dependent on the intracellular ATP level [33–35]. Thus, we also analyzed the proteasome activity of neuronal cells cocultured with MNPs@SiO_2_(RITC)-activated microglia (Fig. 4c). In line with the decrease in intracellular ATP (Fig. 4d-f), proteasome activities were decreased in neuronal cells cocultured with 100 µg/ml MNPs@SiO_2_(RITC)-activated microglia and the decrements were also alleviated in the presence of DCKA (Fig. 4g-i). In addition, we evaluated the effects of D-serine on the intracellular ATP level and proteasome activity in D-serine-treated SH-SY5Y cells, dopaminergic neurons, and cortical neurons (Supplementary Fig. ﻿11). Decreases in the intracellular ATP level and proteasome activity were more pronounced in cortical neurons than in dopaminergic neurons; these decreases were alleviated in the presence of DCKA. However, there were no significant changes in D-serine-treated SH-SY5Y cells. The impairments of mitochondria and proteasome activity in the neuronal cells were highly correlated with exposure to D-serine secreted from the MNPs@SiO_2_(RITC)-activated microglia.

### Inclusion body formation in neuronal cells cocultured with microglia

**Fig. 5:**
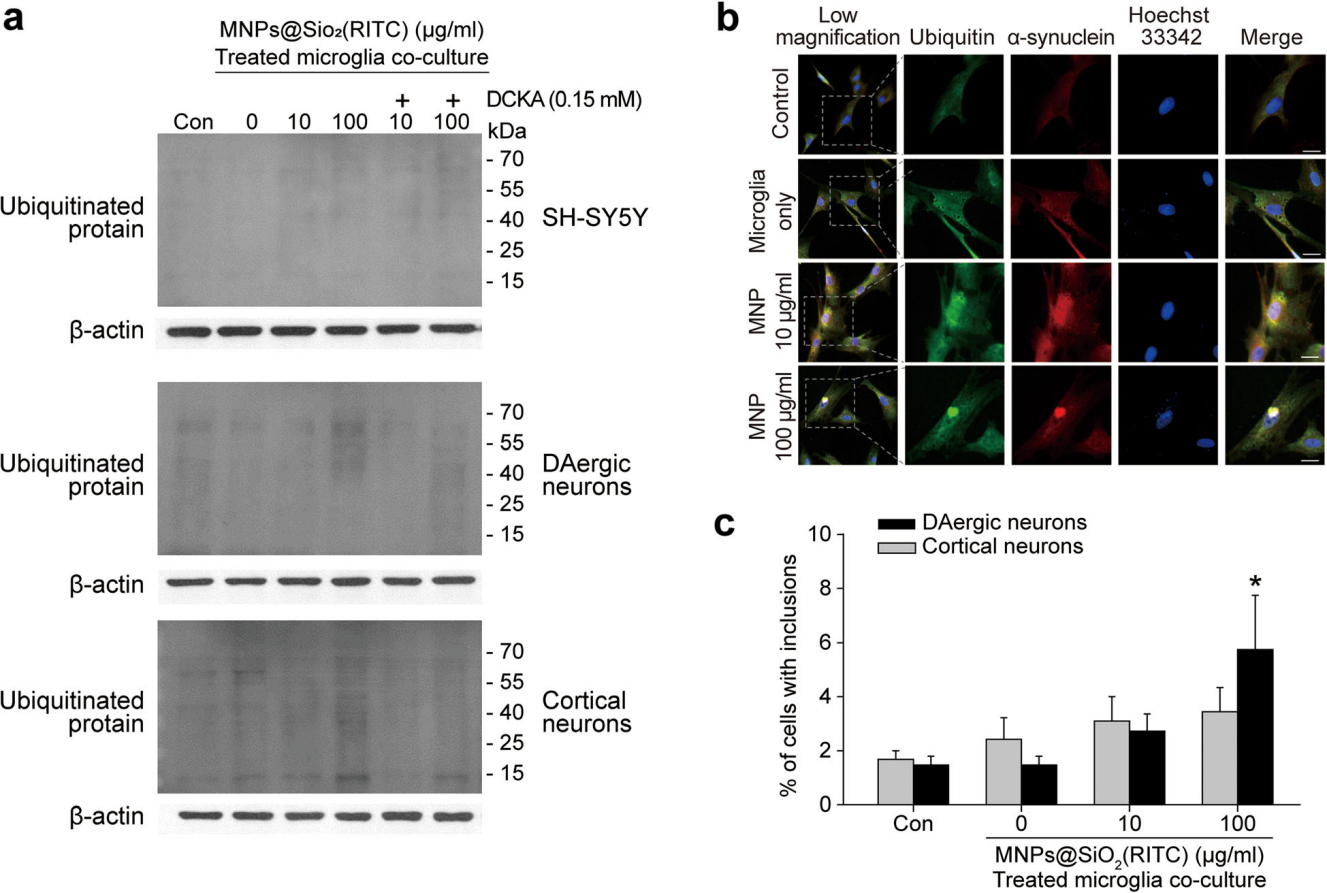
Ubiquitinated proteins and inclusion bodies accumulate in MNPs@SiO_2_(RITC)-treated microglia-cocultured neurons.** a** Immunoblotting analysis of ubiquitinated proteins in the MNPs@SiO_2_(RITC)-treated microglia-cocultured neurons. **b** Inclusion body formation in MNPs@SiO_2_(RITC)-treated microglia-cocultured cortical neurons. Ubiquitin (green), α-synuclein (red), and nucleus (blue) were immunostained. Scale bar = 10 μm. **c** Quantification (%) of cells with inclusion bodies. *p < 0.05 vs. non-treated control

We assessed the accumulation of ubiquitinated proteins following the reduction in proteasome activity. Ubiquitinated protein levels were significantly higher in neuronal cells cocultured with MNPs@SiO_2_(RITC)-activated microglia (Fig. 5a). When neuronal cells were cocultured with the 100 µg/ml MNPs@SiO_2_(RITC)-activated microglia, the increase in ubiquitinated proteins was the most pronounced in the cortical neurons. DCKA suppressed the increases in ubiquitinated proteins.

Immunocytochemical analysis revealed a dramatic increase in inclusion bodies in cortical neuronal cells cocultured with 100 µg/ml MNPs@SiO_2_(RITC)-activated microglia (Fig. 5b). However, inclusion body formation was not detected in SH-SY5Y cells (Supplementary Fig. 12). We observed a dose-dependent increase in inclusion bodies in the DAergic neuronal cells cocultured with MNPs@SiO_2_(RITC)-activated microglia (Supplementary Fig. 13), but not as strong as that observed in the cortical neuronal cells (Fig. 5c).

### Microglial activation and D-serine secretion in MNPs@SiO_2_(RITC)-treated microglia *in vivo*

**Fig. 6:**
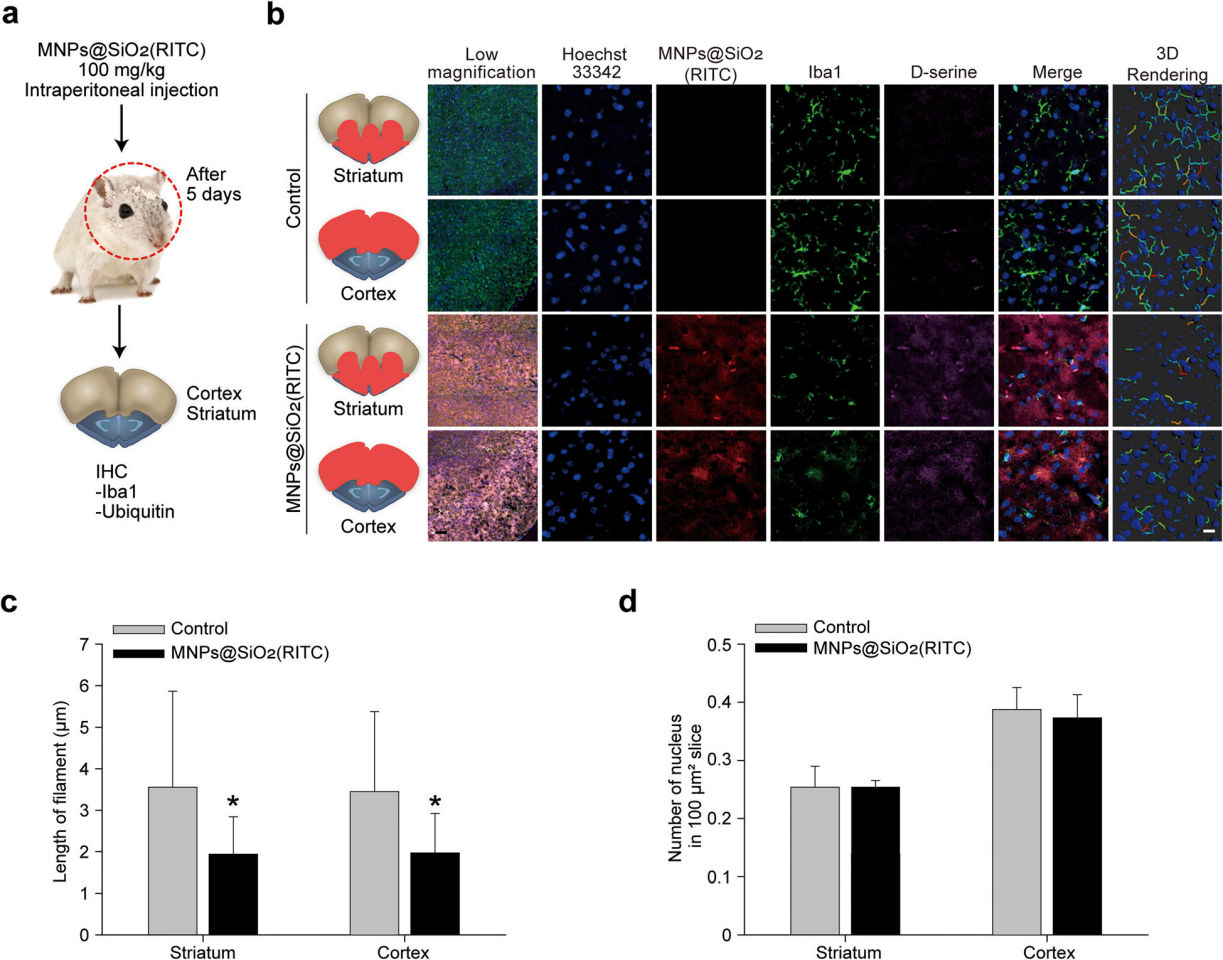
Microglial activation and D-serine distribution in MNPs@SiO_2_(RITC)-treated mouse brains.**a** Schematic representation of the *in vivo* analysis. **b** Immunohistochemical analysis of the cerebellum regions of mice. Low-magnification images are merged with the florescence images of Hoechst 33,342 (blue), MNPs@SiO_2_(RITC) (red), Iba1 (green), and D-serine (violet) to show region-specific structures and the distribution of MNPs@SiO_2_(RITC) and D-serine. Scale bar = 100 μm. Magnified images are separated into Hoechst 33,342, MNPs@SiO_2_(RITC), Iba1, and D-serine and Iba1-based 3D-rendered images. White scale bar = 10 μm. **c** Filament length determined from the 3D-rendered images. **d** Quantification of nuclei in brain sections. *p < 0.05 vs. control

To assess MNPs@SiO_2_(RITC) nanotoxicity on microglia *in vivo*, mice were intraperitoneally injected with MNPs@SiO_2_(RITC) for 5 days. Mouse brains were collected and divided into cortex and striatum for immunohistochemical analysis (Fig. 6a, b). MNPs@SiO_2_(RITC) were homogeneously distributed in the brain and preferentially accumulated in Iba1-positive microglia. Moreover, MNPs@SiO_2_(RITC)- and Iba1-positive microglia showed an activated morphology. D-Serine was distributed around the activated microglia. A 3D reconstruction of microglia morphology clearly showed that the branched structures of microglia were significantly shortened in MNPs@SiO_2_(RITC)-treated mice (Fig. 6c). Cell numbers were not affected (Fig. 6d); however, ubiquitin aggresome-like cell structures, which contain MNPs@SiO_2_(RITC) but not MNPs@SiO_2_(RITC), were detected in the striatum and cortex of MNPs@SiO_2_(RITC)-treated mice (Supplementary Fig. 14).

## Discussion

The present study combined conventional biomolecular and integrated omics-based analyses to evaluate the MNPs@SiO_2_(RITC)-induced activation of microglia and the neuronal toxicity of the activated microglia. Especially, we profiled the transcriptome and amino acids in the MNPs@SiO_2_(RITC)-treated microglia and subjected significantly changed factors to functional analysis to deduce relationships between microglial activation and D-serine-related mechanisms after NP exposure. We demonstrated that high-concentration MNPs@SiO_2_(RITC) induced microglial activation and D-serine secretion. The high concentration of D-serine secreted from the activated microglia induced neuronal cell death, especially in cortical neurons, *via* excitotoxicity, including mitochondrial depolarization, a reduction in intracellular ATP, a decrease in proteasomal activity, and the accumulation of ubiquitinated proteins. Our results suggested that the secondary effect of nanoparticle exposure should also be considered in toxicological assessments. Moreover, minimizing NP usage for drug delivery, imaging, and diagnosis purposes is important for preventing nanotoxicity in the brain with respect to microglial activation and neuronal excitotoxicity.

The direct effect of MNPs@SiO_2_(RITC) on microglia should also be considered. ROS generation by NPs triggered mitochondrial dysfunction and decreased ATP production [9, 11, 36]. Owing to mitochondrial dysfunction, microglia exposed to NPs might exhibit limited regulation of brain homeostasis, which is a decisive role of the microglia in the brain. Therefore, toxicological assessment of microglia exposed to MNPs@SiO_2_(RITC) is also necessary for understanding the neuronanotoxicity of MNPs@SiO_2_(RITC).

In this study, MNPs@SiO_2_(RITC) was used in a monodispersed state, as shown in Supplementary Fig. 1. However, the parameters of NPs reaching the brain depend on the form of the primary particle, agglomerates, and aggregates; therefore, these factors should be considered [37, 38]. Thus, further studies are required to determine the level of NP agglomeration and aggregation for assessing neuronanotoxicity in the brain.

The leaching of MNPs@SiO_2_(RITC) components, including RITC and cobalt ferrite, has previously been considered during long-term exposure. In a previous study, the fluorescence intensities of RITC were decreased by 10 % at the third passage and by 60 % at the seventh passage in human cord blood-derived mesenchymal stem cells; this phenomenon was considered a dilution effect by cell proliferation [10]. In addition, cobalt ferrite is highly toxic [8, 9]; however, there were no significant decrease in the viability of MNPs@SiO_2_(RITC)-treated HEK293 cells for seven days. Moreover, MNPs@SiO_2_(RITC)-treated mice did not show any pathological symptoms until four weeks [5]. Thus, we postulated that the MNPs@SiO_2_(RITC) remained intact for at least seven days and that the component leaching effect may be minor in MNPs@SiO_2_(RITC)-treated cells. However, the subtle toxicity caused by leaching of MNPs@SiO_2_(RITC) components should be considered during the long-term exposure of MNPs@SiO_2_(RITC) and requires further analysis.

We observed intracellular granular structure formation and increment of D-serine secretion in MNPs@SiO_2_(RITC)-treated microglia. D-Serine secretion in microglia responds to amyloid beta, LPS, and secreted amyloid precursor protein [39, 40], but D-serine secretion in NP-treated microglia had not been reported. Concomitant with the increase in D-serine secretion, gene expression related to the transport of serine family amino acids was increased as indicated by combined transcriptome and amino acid profiling analyses. In astrocytes, D-serine secretion mechanisms include synaptosomal-associated proteins receptor (SNARE)-dependent exocytosis as well as non-exocytic mechanisms such as volume-regulated anion channel, alanine–serine–cysteine transporter, and gap junction hemichannels. The mechanism of D-serine release by microglia is still unclear [40, 41]. Moreover, although we have previously observed excitotoxic glutamate production in MNPs@SiO_2_(RITC)-treated HEK293 cells based on metabotranscriptome analysis [9], the mechanisms of D-serine production and secretion in MNPs@SiO_2_(RITC)-treated microglia have not been unraveled. In MNPs@SiO_2_(RITC)-treated microglia, D-serine did not colocalize with VAMP2 nor NPY, which are exocytic vesicle markers in astrocytes (Supplementary Fig. 15) [42, 43], suggesting that microglia utilize a pathway for D-serine secretion different from that of astrocytes and detailed future analysis is required to reveal the specific mechanism.

In a previous study, the MNPs@SiO_2_(RITC)-uptake efficiency of MCF-7 mammary gland adenocarcinoma cells was determined using ICP-atomic emission spectroscopy [6]. In addition, MCF-7 cells have been thoroughly characterized in nanoparticle uptake studies with various composites of nanoparticles and are thought to be a candidate for model cell lines [44, 45]. The efficiency was approximately 10^5^ particles/cell at 2.0 mg/ml MNPs@SiO_2_(RITC) in MNPs@SiO_2_(RITC)-treated MCF-7 cells. In the case of BV2 cells in this study, the efficiency of internalized MNPs@SiO_2_(RITC) was approximately 2.3 × 10^4^ particles/cell at 10 µg/ml MNPs@SiO_2_(RITC) and 2.0 × 10^5^ particles/cell at 100 µg/ml MNPs@SiO_2_(RITC). Thus, the MNPs@SiO_2_(RITC)-uptake efficiency of BV2 cells was approximately 40-fold higher than that of MCF7 cells.

The MNPs@SiO_2_(RITC)-induced D-serine secretion reduced the intracellular ATP level, mitochondrial function, and proteasomal activity in neuronal cells *via* excitotoxicity. Excitotoxicity is highly correlated with the pathological phenotype of neurodegenerative disease [46, 47], and it is triggered by the opening of calcium channels, including the NMDA receptor, by agonists. Physiologically, extracellular and intracellular calcium ion concentrations differ by 20,000-fold (mM vs. 100 nM) [48]. The excessive influx of calcium ions is regulated by storage in the endoplasmic reticulum and mitochondria in the physiological state [49]. However, an overwhelming influx of calcium ions against the physiological regulation induces a calcium ion imbalance [50] and activates calcium ion-cofactor catabolic enzyme activities, which are involved in cell death mechanisms [49]. Moreover, overloaded calcium ions induce mitochondrial membrane depolarization, mitochondrial dysfunction, and reduced ATP production [30, 51]. ATP is essential for ubiquitination, de-ubiquitination, and proteasome complex assembly [33–35]. When ATP strongly decreases, the ubiquitin-proteasome system is corrupted, resulting in the aggregation and accumulation of defective proteins in cells. In neurons, such protein aggregations are histopathological biomarkers for neurodegenerative diseases [52]. Thus, the D-serine level in the brain may be monitored after NP exposure as a secondary (indirect) phenotype of neuronanotoxicity.

Cortical neuronal cells reacted more sensitively to D-serine-induced excitotoxicity than DAergic neurons. Overactivation of the NMDA receptor by D-serine induces neuronal death and protein aggregation [53, 54]. In this study, cortical Lewy bodies, which are a pathological feature of Lewy body dementia closely related to Parkinson’s disease [55], were detected in in vitro MNPs@SiO_2_(RITC)-activated microglia-cocultured cortical neurons and *in vivo* in the mouse brain. Neuronal cells in the MNPs@SiO_2_(RITC)-activated microglia coculture system showed decreased viability, along with decreases in intracellular ATP and proteasome activity, and the effects were the most pronounced in cortical neurons. Our previous study showed that DAergic neurons are more vulnerable to MNPs@SiO_2_(RITC)-induced nanotoxicity (direct effect) than cortical neuronal cells [8]. In this study, cortical neurons responded more sensitively to D-serine secreted from MNPs@SiO_2_(RITC)-activated microglia than DAergic neurons. This is consistent with the finding that cortical neurons are more vulnerable to excitotoxic conditions than DAergic neurons, because endogenous dopamine from DAergic neurons can block excitotoxicity-induced calcium signals and plays a major role in conserving calcium homeostasis in cells [56]. Moreover, molecules abundant in DAergic neurons, including kynurenic acid, brain-derived neurotrophic factor, and prostaglandin A1, protect against excitotoxicity in DAergic neurons [57–59]. Collectively, we showed the induction of a series of dysfunctions in neuronal cells by D-serine secreted from MNPs@SiO_2_(RITC)-activated microglia, suggesting the secondary toxicity of NPs.

In a previous study, the mouse whole-body biodistribution of MNPs@SiO_2_(RITC) was investigated at three different concentrations (25 mg/kg, 50 mg/kg, and 100 mg/kg) using a confocal microscope [5]. Intraperitoneally administered MNPs@SiO_2_(RITC) accumulated in the brain, spleen, heart, liver, lungs, kidneys, and uterus. Although quantification of MNPs@SiO_2_(RITC) in the brain was limited, the fluorescence intensity of cells was similar between brain slices of mice receiving 100 mg/kg and microglia treated with 10 µg/ml MNPs@SiO_2_(RITC). To compare the relevance of NPs *in vitro versus* that *in vivo* in the brain, further research is required to calculate the number of NPs *in vivo* using high-resolution techniques such as X-ray-based analytics, magnetic resonance imaging, or laser ablation ICP-MS [60–62]. In addition, further studies are required to investigate the interspecies comparisons and the possibility of using cell lines *versus* primary cultures in NP-treated cells.

## Conclusions

Based on transcriptomics and amino acid profiling, this study revealed that NP-induced microglial activation is associated with excitotoxic D-serine secretion. The present findings suggest that exposure to NPs can directly activate microglia and indirectly be deleterious to neurons. These results highlight the importance of the safety evaluation of NPs and the assessment of secondary nanotoxicity effects.

## Supplementary Information

**Additional file 1: Supplementary Table 1.** Quantitative real-time PCR primer sequences for genes encoding transcriptomic network related genes.

**Additional file 2: Supplementary Table 2.** Quantitative real-time PCR primer sequences for genes encoding combined transcriptome and amino acid profiles network related genes.

**Additional file 3: Supplementary Table 3.** Ingenuity Pathway Analysis-based profiles of transcriptome of BV2 cells treated with MNPs*@*SiO_2_(RITC).

**Additional file 4: Supplementary Table 4.** Amino acid amount in cell mass of MNPs@SiO_2_(RITC)-treated BV2 cells.

**Additional file 5: Supplementary Table 5.** Ingenuity Pathway Analysis-based profiles of transcriptome and amino acids of BV2 cells treated with MNPs*@*SiO_2_(RITC).

**Additional file 6: Supplementary Table 6. **Concentrations of secreted amino acids in media of MNPs@SiO_2_(RITC)-treated BV2 cells.

**Additional file 7: Supplementary Figure 1.** Transmission electron microscope images for **a﻿** MNPs@SiO_2_(RITC) and **﻿b** SiO_2_ NPs. Scale bar = 20 nm.

**Additional file 8: Supplementary Figure 2.** XRD spectrum of MNPs@SiO_2_(RITC). Peaks in 30^o^, 36^o^, 44^o^, 57^o^ and 64 ^o^ are specific patterns of cobalt ferrite and the broad peak between 20^o^ and 40^o^ indicate the amorphous silica beads.

**Additional file 9: Supplementary Figure 3.** Characterization of primary rat neuronal cells and microglia. **a﻿** Immunofluorescence of cortical neurons stained with anti-NeuN antibody. **b﻿** Immunofluorescence of DAergic neurons stained with anti-TH antibody. Scale bar = 10 μm. **c﻿** Immunofluorescence of cortical and DAergic neurons stained with anti-MAP2 and DAT antibodies. Scale bar = 20 μm. **d﻿** FACS analysis for OX-42 expression in primary rat microglia. Percentage indicates positively stained population of cells.

**Additional file 10: Supplementary Figure 4.** Microglia activation after MNPs@SiO_2_(RITC) treatment. Morphological analysis of **a﻿** BV2 and **b﻿** primary rat microglia. Scale bar = 50 μm. Red; MNPs@SiO_2_(RITC).

**Additional file 11: Supplementary Figure 5.** Microglia activation after SiO_2_ NPs treatment. Morphological analysis of BV2 and primary rat microglia. Scale bar = 50 μm.

**Additional file 12: Supplementary Figure 6.** RNA-seq-based transcriptome analysis of BV2 cells treated with MNPs@SiO_2_(RITC) for 12 h. **﻿a** Categorization of DEGs identified by RNA-seq. DEGs were analyzed using the MGI GO tool. Metabolism-related genes are highlighted. **b﻿** Heat map of DEGs (fold change > 1.5 or <–1.5), including 29 DEGs related to metabolism (left panel) and 18 DEGs related to inflammation (right panel), in control cells and 100 and 10 µg/ml MNPs@SiO_2_(RITC)-treated cells. **﻿c** Metabolism- and inflammation-related gene network for 100 µg/ml-treated BV2 cells constructed by IPA. The red and green areas indicate the up- and downregulated genes, respectively.

**Additional file 13: Supplementary Figure 7.** Transcriptomic network of 10 µg/ml treated BV2 cells. Metabolism and inflammation related genes and network of 10 µg/ml treated BV2 cells was constructed algorithmically by IPA. Red and green areas indicate up- and downregulated genes, respectively.

**Additional file 14: Supplementary Figure 8.** Combinatorial analysis of RNA-seq and intracellular amino acid profiles 10 µg/ml MNPs@SiO_2_(RITC) treated BV2. Transcriptome combined with amino acids profiles network were constructed algorithmically by IPA in 10 µg/ml MNPs@SiO_2_(RITC) treated BV2. Red and green areas indicate up- and downregulated genes, respectively. Cut off fold change ± 1.5 for genes and ± 1.2 for amino acids were used.

**Additional file 15: Supplementary Figure 9.** Evaluation of cytotoxicity on neuronal cells in coculture system with activated microglia. **﻿a** Morphological analysis and cell density observation of SH-SY5Y, DAergic, and cortical neurons. Scale bar = 50 μm. **﻿b** MTS assay for evaluation of cocultured neurons viability. Data were normalized with non-treated control and represent mean ± SD of three independent experiments. *p < 0.05 vs. non-treated control.

**Additional file 16: Supplementary Figure 10.** Evaluation of changes in mitochondria membrane potential in MNPs@SiO_2_(RITC) treated primary rat microglia cocultured neuronal cells. Mitochondria membrane potentials were analyzed using 5,5’,6,6’-tetrachloro-1,1’,3,3’- tetraethylbenzimidazolyl carbocyanine iodide (JC-1). Red fluorescence (high membrane potential) and green fluorescence (low membrane potential) were measured by FACS. Percentage indicates the portion of cells with low mitochondria membrane potential. Carbonyl cyanide *m*-chlorophenyl hydrazine (CCCP) were used as positive control for decreasing mitochondria membrane potential.

**Additional file 17: Supplementary Figure 11.** Evaluation of the intracellular ATP level and proteasomal activity in D-serine treated neuronal cells. Luminescence images of the ATP level and proteasomal activity for **﻿a** SH-SY5Y, **b﻿** DAergic neurons, and **c﻿** cortical neurons. Statistical analysis of ATP levels in **﻿d** SH-SY5Y, **e﻿** DAergic, and f cortical neurons. Statistical analysis of proteasomal activity in g SH-SY5Y, h DAergic, and i cortical neurons. *p < 0.05 vs. without DCKA. Con: neuronal cells only. The experiments were repeated three times independently, and a representative result is shown.

**Additional file 18: Supplementary Figure 12.** Immunocytochemistry analysis in MNPs@SiO_2_(RITC) treated microglia cocultured SH-SY5Y cells. Images were acquired by immunostaining with ubiquitin (green), α-synuclein (red), and nucleus (blue) Scale bar = 10 μm.

**Additional file 19: Supplementary Figure 13.** Inclusion body formation in MNPs@SiO_2_(RITC) treated microglia cocultured DAergic neurons. Images of inclusion body formation were acquired by immunostaining with ubiquitin (green), α-synuclein (red), and nucleus (blue) Scale bar = 10 μm.

**Additional file 20: Supplementary Figure 14.** Inclusion body formation in MNPs@SiO_2_(RITC) treated mice brains. Images of inclusion body formation were acquired by immunostaining with ubiquitin (green), MNPs@SiO_2_(RITC) (red), and nucleus (blue). Black scale bar = 100 μm. White scale bar = 10 μm. White arrowhead indicates inclusion body without colocalized to MNPs@SiO_2_(RITC) and black arrowhead indicates inclusion body with colocalized to MNPs@SiO_2_(RITC).

**Additional file 21: Supplementary Figure 15.** Immunostaining analysis for exocytic vesicle marker and D-serine﻿ in MNPs@SiO_2_(RITC) treated microglia. Images of exocytic vesicle marker and D-serine﻿ were acquired by immunostaining with MNPs@SiO_2_(RITC) (red), VAMP2 or NPY (green), D-serine﻿ (violet), and nucleus (blue). Stained with VAMP2 in **﻿a** BV2 and **﻿b** primary rat microglia and stained with NPY in **﻿c** BV2 and **d﻿** primary rat microglia were analysed. Scale bar = 20 μm.

## Data Availability

The data supporting the findings of this study are available from the corresponding author, upon reasonable request. Transcriptome sequencing and quantification data are available in the GEO database under the following accession number: GSE154250.

## Abbreviations

Ahr: Aryl-hydrocarbon receptor
ANOVA: Analysis of variance
BBB: Blood–brain barrier
*Cbs/Cbsl*: Cystathionine beta-synthase
CCCP: Carbonyl cyanide m-chlorophenyl hydrazine
*Ccr1*: Chemokine (C-C motif) receptor 1
CNS: Central nervous system
CoFe_2_O_3_: Cobalt ferrite
*Csf3*: Colony-stimulating factor 3
*Cxcl3*: Chemokine (C-X-C motif) ligand 3
DAergic: Dopaminergic
DAT: Dopamine transporter
DCKA: 5,7-dichlorokynurenic acid
DEG: Differentially expressed gene
DsdSC: D-serine dehydratase from *Saccharomyces cerevisiae*
EOC: Ethoxycarbonyl
FACS: Fluorescence-activated cell sorting
*Fpr1*: Formyl peptide receptor 1
GC: Gas chromatography
GO: Gene ontology
HF: Hydrogen fluoride
ICP-MS: Inductively coupled plasma mass spectrometry
IPA: Ingenuity pathway analysis
JC-1: 5,5',6,6'-tetrachloro-1,1',3,3'- tetraethylbenzimidazolyl carbocyanine iodide
LC: Liquid chromatography
LDH: Lactate dehydrogenase
MAP2: Microtubule-associated protein 2
MGI: Mouse genome informatics
MNPs: Magnetic nanoparticles
*Mylk*: 130-kDa myosin light chain kinase
NADH: Nicotinamide adenine dinucleotide
NeuN: Neuronal nuclei
NMDA: *N*-methyl-D-aspartate
NO: Nitric oxide
NPs: Nanoparticles
NPY: Neuropeptide Y
PFA: Paraformaldehyde
RITC: Rhodamine B isothiocyanate
ROS: Reactive oxygen species
RT: Room temperature
RT-qPCR: Quantitative reverse transcription PCR
*Serpinf1*: Serine (or cysteine) peptidase inhibitor, clade F, member 1
SIM: Selected-ion monitoring
*Slc1a4*: Solute carrier family 1 (glutamate/neutral amino acid transporter), member 4
*Slc6a9*: Solute carrier family 6 (neurotransmitter transporter, glycine), and member 9
SNARE: Synaptosomal-associated proteins receptor
TBDMS: Tert-butyldimethylsilyl
TEM: Transmission electron microscopy
TH: Tyrosine hydroxylase
VAMP2: Vesicle-associated membrane protein 2
XRD: X-ray diffraction
