# Supplementary material for: Silica-coated magnetic nanoparticles activate microglia and induce neurotoxic d-serine secretion
Source: Part Fibre Toxicol. 2021 Aug 12;18:30. doi: 10.1186/s12989-021-00420-3 (PMC8359100; doi:10.1186/s12989-021-00420-3)
Supplement: Supplementary file 1 — Additional file 1: Supplementary Table 1. Quantitative real-time PCR primer sequences for genes encoding transcriptomic network related genes. [file 12989_2021_420_MOESM1_ESM.docx]

**Supplementary Table 1**. Quantitative real-time PCR primer sequences for genes encoding transcriptomic network related genes

| **Gene Name** | **Symbol** | **NCBI Ref. seq** | **Direction** | **Primer sequence (5’-3’)** |
| --- | --- | --- | --- | --- |
| chemokine (C-C motif) receptor 1 | Ccr1 | BC011092.1 | Forward | GAAGCCTACCCCACAACTAC |
|  |  |  | Reverse | AGAATCACTAGGACATTGCCC |
| chemokine (C-X-C motif) ligand 3 | Cxcl3 | BC117014.1 | Forward | TTTGAGACCATCCAGAGCTTG |
|  |  |  | Reverse | CCTTGAGAGTGGCTATGACTTC |
| formyl peptide receptor 1 | Fpr1 | NM_013521.2 | Forward | TCATATCCACAATCCAAGTCCG |
|  |  |  | Reverse | GTCTTTCTCTGAAGTCCTGGC |
| serine (or cysteine) peptidase inhibitor, clade F, member 1 | Serpinf1 | BC019852.1 | Forward | ACATCCACAGCACCTACAAG |
|  |  |  | Reverse | ATTCTGGAAGCACTCTTGAGG |
| glyceraldehyde-3-phosphate dehydrogenase | Gapdh | NM_001289726.1 | Forward | GAAGACTGTGGATGGCCC |
|  |  |  | Reverse | CCATGCCAGTGAGCTTCC |

Ref. seq.: Reference sequence
