# Supplementary material for: Silica-coated magnetic nanoparticles activate microglia and induce neurotoxic d-serine secretion
Source: Part Fibre Toxicol. 2021 Aug 12;18:30. doi: 10.1186/s12989-021-00420-3 (PMC8359100; doi:10.1186/s12989-021-00420-3)
Supplement: Supplementary file 2 — Additional file 2: Supplementary Table 2. Quantitative real-time PCR primer sequences for genes encoding combined transcriptome and amino acid profiles network related genes. [file 12989_2021_420_MOESM2_ESM.docx]

**Supplementary Table 2**. Quantitative real-time PCR primer sequences for genes encoding combined transcriptome and amino acid profiles network related genes

| **Gene Name** | **Symbol** | **NCBI Ref. seq** | **Direction** | **Primer sequence (5’-3’)** |
| --- | --- | --- | --- | --- |
| aryl-hydrocarbon receptor | Ahr | BC040248.1 | Forward | TCCCAGAAACCTCCACAATG |
|  |  |  | Reverse | TGCAGCGTAATGGACATAGG |
| cystathionine beta-synthase (-like protein) | Cbs/Cbsl | BC013480.1 | Forward | GAGTATGGAGAAGGTGGATGTG |
|  |  |  | Reverse | GGGAATCAAATCTGGCATTGG |
| 130 kDa myosin light chain kinase | Mylk | AY237727.2 | Forward | AGAGAAACCTGAAGAGCCAAAG |
|  |  |  | Reverse | AATTTCCCAGATCCTAGTCGC |
| solute carrier family 1 (glutamate/neutral amino acid transporter), member 4 | Sls1a4 | BC043483.1 | Forward | ACCTGTTCCCTTCCAATCTTG |
|  |  |  | Reverse | TGTTCATCCCTTCCACATCTG |
| solute carrier family 6 (neurotransmitter transporter, glycine), member 9 | Sls6a9 | BC021828.1 | Forward | CATCTATGGGCACCGTAACTAC |
|  |  |  | Reverse | CTGGATCACCGTGAAGATGAG |
| glyceraldehyde-3-phosphate dehydrogenase | Gapdh | NM_001289726.1 | Forward | GAAGACTGTGGATGGCCC |
|  |  |  | Reverse | CCATGCCAGTGAGCTTCC |

Ref. seq.: Reference sequence
