# Supplementary material for: Silica-coated magnetic nanoparticles activate microglia and induce neurotoxic d-serine secretion
Source: Part Fibre Toxicol. 2021 Aug 12;18:30. doi: 10.1186/s12989-021-00420-3 (PMC8359100; doi:10.1186/s12989-021-00420-3)
Supplement: Supplementary file 3 — Additional file 3: Supplementary Table 3. Ingenuity Pathway Analysis-based profiles of transcriptome of BV2 cells treated with MNPs@SiO2(RITC). [file 12989_2021_420_MOESM3_ESM.docx]

**Supplementary Table 3**. Ingenuity Pathway Analysis-based profiles of transcriptome of BV2 cells treated with MNPs*@*SiO_2_(RITC)

| Entrez gene name | Symbol | Location | Signal fold change^a^ | |
| --- | --- | --- | --- | --- |
|  |  |  | 10 μg/ml | 100 μg/ml |
| ATP binding cassette subfamily D member 2 | ABCD2 | Cytoplasm | 1.00 | -2.04 |
| arginase 1 | ARG1 | Cytoplasm | 1.00 | -1.84 |
| complement C1q A chain | C1QA | Extracellular Space | 1.00 | -2.20 |
| C-C motif chemokine ligand 2 | CCL2 | Extracellular Space | 1.00 | 2.45 |
| C-C motif chemokine receptor 1 | CCR1 | Plasma Membrane | 1.00 | 1.67 |
| CD83 molecule | CD83 | Plasma Membrane | 1.00 | -2.35 |
| C-type lectin domain family 12 member A | CLEC12A | Plasma Membrane | 1.00 | -1.67 |
| ceruloplasmin (ferroxidase) | CP | Extracellular Space | 1.00 | -3.92 |
| colony stimulating factor 3 | CSF3 | Extracellular Space | 1.00 | 2.25 |
| colony stimulating factor 3 receptor | CSF3R | Plasma Membrane | 1.00 | -1.57 |
| C-X-C motif chemokine ligand 10 | CXCL10 | Extracellular Space | 1.00 | 1.53 |
| C-X-C motif chemokine ligand 3 | CXCL3 | Extracellular Space | 1.68 | 2.64 |
| cytochrome P450 family 11 subfamily A member 1 | CYP11A1 | Cytoplasm | 1.00 | 2.10 |
| DNA damage inducible transcript 4 | DDIT4 | Cytoplasm | 1.00 | -2.13 |
| FosB proto-oncogene, AP-1 transcription factor subunit | FOSB | Nucleus | 0.74 | -2.19 |
| formyl peptide receptor 1 | FPR1 | Plasma Membrane | 1.00 | -4.08 |
| glycerol-3-phosphate acyltransferase 3 | GPAT3 | Cytoplasm |  | 1.56 |
| G protein-coupled receptor 183 | GPR183 | Plasma Membrane | 1.00 | -1.62 |
| haptoglobin | HP | Extracellular Space | 1.00 | -2.62 |
| interleukin 1 receptor type 1 | IL1R1 | Plasma Membrane | 1.17 | -2.01 |
| interleukin 1 receptor antagonist | IL1RN | Extracellular Space | 1.00 | 1.66 |
| integrin subunit alpha L | ITGAL | Plasma Membrane | 1.00 | -2.20 |
| notch 1 | NOTCH1 | Plasma Membrane | 1.00 | -1.72 |
| nuclear protein 1, transcriptional regulator | NUPR1 | Nucleus | 1.00 | -1.87 |
| purinergic receptor P2Y13 | P2RY13 | Plasma Membrane | 1.16 | -2.08 |
| PR domain 1 | PRDM1 | Nucleus | 1.12 | -1.51 |
| Rab interacting lysosomal protein | RILP | Cytoplasm | 1.35 | 1.88 |
| serpin family F member 1 | SERPINF1 | Extracellular Space | 1.08 | -1.56 |
| sialic acid binding Ig like lectin 9 | SIGLEC9 | Plasma Membrane | 1.00 | -1.95 |
| solute carrier family 13 member 3 | SLC13A3 | Plasma Membrane | 1.02 | -1.75 |
| solute carrier family 36 member 2 | SLC36A2 | Plasma Membrane | 1.00 | -2.50 |
| thromboxane A synthase 1 | TBXAS1 | Plasma Membrane | 1.06 | -1.56 |
| toll-like receptor 12 | TLR12 | Other | -1.80 | -2.36 |
| TNF receptor superfamily member 4 | TNFRSF4 | Plasma Membrane | 1.65 | 1.77 |
| tribbles pseudokinase 3 | TRIB3 | Nucleus | 1.00 | -1.64 |
| thioredoxin interacting protein | TXNIP | Cytoplasm | 1.00 | 1.66 |

^a^Normalized signal fold change of signal in treated groups with MNPs@SiO_2_(RITC) to corresponding signal of in control group
