# Supplementary material for: Silica-coated magnetic nanoparticles activate microglia and induce neurotoxic d-serine secretion
Source: Part Fibre Toxicol. 2021 Aug 12;18:30. doi: 10.1186/s12989-021-00420-3 (PMC8359100; doi:10.1186/s12989-021-00420-3)
Supplement: Supplementary file 4 — Additional file 4: Supplementary Table 4. Amino acid amount in cell mass of MNPs@SiO2(RITC)-treated BV2 cells. [file 12989_2021_420_MOESM4_ESM.docx]

**Supplemenatary Table 4.** Amino acid amount in cell mass of MNPs@SiO_2_(RITC)-treated BV2 cells

|  |  | amount (ng/4 × 10^5^ cells, ± standard deviation)^a^ | | |  |  |  |
| --- | --- | --- | --- | --- | --- | --- | --- |
|  |  |  | MNPs@SiO_2_(RITC)-treated group (n = 3) | |  | Ratio value^e^ | |
| No. | Analyte | Control  (n = 3) | 10 µg/ml | 100 µg/ml | *p* value^d^ | 10  µg/ml | 100 µg/ml |
| 1 | Alanine | 44.7 ± 3.0 | 48.7 ± 5.1 (0.547)^b^ | 51.5 ± 5.1 (0.232)^c^ | 0.745 | 1.09 | 1.15 |
| 2 | Glycine | 80.8 ± 5.1 | 80.9 ± 10.7 (1.000) | 80.3 ± 5.0 (0.996) | 0.995 | 1.00 | 0.99 |
| 3 | Valine | 14.8 ± 0.8 | 15.1 ± 0.6 (0.962) | 17.4 ± 1.6 (0.065) | 0.092 | 1.02 | 1.17 |
| 4 | Leucine | 20.8 ± 0.7 | 20.5 ± 0.2 (0.922) | 25.0 ± 1.7 (0.007) | 0.005 | 0.98 | 1.20 |
| 5 | Isoleucine | 18.2 ± 0.8 | 18.5 ± 0.4 (0.972) | 22.1 ± 2.6 (0.059) | 0.078 | 1.02 | 1.21 |
| 6 | Proline | 51.2 ± 3.5 | 54.5 ± 5.7 (0.621) | 46.9 ± 2.4 (0.446) | 0.137 | 1.06 | 0.91 |
| 7 | Pyroglutamic acid | 189.2 ± 12.8 | 168.6 ± 27.7 (0.422) | 120.9 ± 10.9 (0.010) | 0.047 | 0.89 | 0.64 |
| 8 | 4-Hydroxyproline | 1.5 ± 0.3 | 1.9 ± 0.3 (0.140) | 1.6 ± 0.1 (0.679) | 0.405 | 1.25 | 1.06 |
| 9 | Serine | 28.6 ± 4.3 | 36.9 ± 11.9 (0.480) | 40.4 ± 6.5 (0.262) | 0.863 | 1.29 | 1.41 |
| 10 | Threonine | 18.8 ± 3.7 | 55.4 ± 57.0 (0.448) | 41.8 ± 17.8 (0.709) | 0.882 | 2.94 | 2.22 |
| 11 | r-Aminobutric acid | 6.3 ± 0.9 | 6.9 ± 1.5 (0.881) | 6.7 ± 1.8 (0.948) | 0.984 | 1.09 | 1.06 |
| 12 | Phenylalanine | 12.4 ± 0.1 | 12.0 ± 0.8 (0.616) | 14.8 ± 0.3 (0.003) | 0.001 | 0.97 | 1.19 |
| 13 | Cysteine | 6.2 ± 1.2 | 6.9 ± 0.6 (0.771) | 10.4 ± 1.6 (0.013) | 0.029 | 1.11 | 1.67 |
| 14 | Aspartic acid | 65.1 ±5.5 | 82.8 ± 10.4 (0.205) | 86.5 ± 15.3 (0.124) | 0.917 | 1.27 | 1.33 |
| 15 | Glutamic acid | 145.1 ± 8.8 | 171.6 ± 35.1 (0.505) | 171.5 ± 31.0 (0.508) | 1.000 | 1.18 | 1.18 |
| 16 | Asparagine | 60.9 ± 19.4 | 117.9 ± 46.5 (0.174) | 141.7 ± 28.7 (0.058) | 0.677 | 1.94 | 2.33 |
| 17 | Glutamine | 11.5 ± 1.7 | 17.5 ± 2.7 (0.339) | 25.7 ± 7.5 (0.024) | 0.161 | 1.51 | 2.22 |
| 18 | Lysine | 39.9 ± 6.1 | 55.3 ± 8.7 (0.275) | 101.9 ± 15.9 (0.001) | 0.005 | 1.39 | 2.56 |
| 19 | Tryptophane | 8.2 ± 1.1 | 12.5 ± 2.3 (0.327) | 19.5 ± 5.3 (0.016) | 0.103 | 1.53 | 2.38 |

^a^Values as each analyte amount (ng/4 × 10^5^ cells). ^b^One way ANOVA comparing the mean values of control group and treated group with MNPs@SiO_2_(RITC) of 10 μg/ml. ^c^One way ANOVA comparing the mean values of control group and treated group with MNPs@SiO_2_(RITC) of 100 μg/ml. ^d^One way ANOVA comparing the mean values of group with MNPs@SiO_2_(RITC) of 10 μg/ml and treated group with MNPs@SiO_2_(RITC) of 100 μg/ml. ^e^Ratio values of analyte in treated groups with MNPs@SiO_2_(RITC) to corresponding mean values in the control group.
