# Supplementary material for: Silica-coated magnetic nanoparticles activate microglia and induce neurotoxic d-serine secretion
Source: Part Fibre Toxicol. 2021 Aug 12;18:30. doi: 10.1186/s12989-021-00420-3 (PMC8359100; doi:10.1186/s12989-021-00420-3)
Supplement: Supplementary file 5 — Additional file 5: Supplementary Table 5. Ingenuity Pathway Analysis-based profiles of transcriptome and amino acids of BV2 cells treated with MNPs@SiO2(RITC). [file 12989_2021_420_MOESM5_ESM.docx]

**Supplementary Table 5**. Ingenuity Pathway Analysis-based profiles of transcriptome and amino acids of BV2 cells treated with MNPs*@*SiO_2_(RITC)

| Entrez gene name | Symbol | Location | Signal fold change^a^ | |
| --- | --- | --- | --- | --- |
|  |  |  | 10 μg/ml | 100 μg/ml |
| aryl hydrocarbon receptor | AHR | Nucleus | 2.22 | 3.46 |
| cystathionine-beta-synthase | CBS/CBSL | Cytoplasm | 2.06 | 2.79 |
| growth hormone inducible transmembrane protein | GHITM | Cytoplasm | -1.04 | -1.26 |
| G protein-coupled receptor kinase 1 | GRK1 | Plasma Membrane | 6.23 | 2.04 |
| mitogen-activated protein kinase 10 | MAPK10 | Cytoplasm | -1.01 | 3.25 |
| myosin light chain kinase | MYLK | Cytoplasm | 1.28 | 1.30 |
| purinergic receptor P2Y12 | P2RY12 | Plasma Membrane | -1.27 | -1.39 |
| purinergic receptor P2Y13 | P2RY13 | Plasma Membrane | 1.08 | -2.08 |
| phosphoenolpyruvate carboxykinase 2, mitochondrial | PCK2 | Cytoplasm | -1.06 | -1.77 |
| serine incorporator 2 | SERINC2 | Plasma Membrane | -2.46 | -4.72 |
| serum/glucocorticoid regulated kinase 1 | SGK1 | Cytoplasm | -1.05 | -1.45 |
| solute carrier family 1 member 4 | SLC1A4 | Plasma Membrane | -1.03 | -1.52 |
| solute carrier family 36 member 2 | SLC36A2 | Plasma Membrane | -1.06 | -2.50 |
| solute carrier family 39 member 4 | SLC39A4 | Plasma Membrane | 1.01 | -1.31 |
| solute carrier family 6 member 9 | SLC6A9 | Plasma Membrane | 1.01 | -1.40 |
| SRC proto-oncogene, non-receptor tyrosine kinase | SRC | Cytoplasm | 1.04 | 1.42 |
| teneurin transmembrane protein 1 | TENM1 | Plasma Membrane | 2.03 | 2.04 |
|  | asparagine | Other | 1.93 | 2.46 |
|  | aspartic acid | Other | 1.27 | 1.31 |
|  | cysteine | Other | 1.04 | 1.55 |
|  | glutamine | Other | 1.47 | 2.07 |
|  | lysine | Other | 1.28 | 2.31 |
|  | serine | Other | 1.16 | 1.27 |
|  | threonine | Other | 2.25 | 1.79 |

^a^Normalized signal fold change of signal in treated groups with MNPs@SiO_2_(RITC) to corresponding signal of in control group
