# Supplementary material for: Silica-coated magnetic nanoparticles activate microglia and induce neurotoxic d-serine secretion
Source: Part Fibre Toxicol. 2021 Aug 12;18:30. doi: 10.1186/s12989-021-00420-3 (PMC8359100; doi:10.1186/s12989-021-00420-3)
Supplement: Supplementary file 6 — Additional file 6: Supplementary Table 6. Concentrations of secreted amino acids in media of MNPs@SiO2(RITC)-treated BV2 cells. [file 12989_2021_420_MOESM6_ESM.docx]

**Supplemenatary Table 6.** Concentrations of secreted amino acids in media of MNPs@SiO_2_(RITC)-treated BV2 cells

|  |  | concentration (μg/ml, ± standard deviation)^a^ | | |  |  |  |
| --- | --- | --- | --- | --- | --- | --- | --- |
|  |  |  | MNPs@SiO_2_(RITC)-treated group (n = 3) | |  | Ratio value^e^ | |
| No. | Analyte | Control  (n = 3) | 10 µg/ml | 100 µg/ml | *p* value^d^ | 10  µg/ml | 100 µg/ml |
| 1 | Alanine | 46.93 ± 3.93 | 44.99 ± 1.79 (0.819)^b^ | 47.39 ± 3.83 (0.386)^c^ | 0.183 | 1.09 | 1.15 |
| 2 | Glycine | 44.44 ± 2.05 | 42.95 ± 1.47 (0.842) | 35.7 ± 2.86 (<0.001) | <0.001 | 0.97 | 0.80 |
| 3 | Valine | 22.35 ± 1.15 | 21.81 ± 0.95 (0.861) | 23.40 ± 0.70 (0.006) | 0.011 | 1.02 | 1.17 |
| 4 | Leucine | 22.89 ± 1.50 | 22.53 ± 1.51 (0.959) | 23.68 ± 1.09 (0.288) | 0.205 | 0.98 | 1.20 |
| 5 | Isoleucine | 52.86 ± 3.37 | 50.74 ± 2.46 (0.655) | 54.6 ± 1.28 (<0.001) | <0.001 | 1.02 | 1.21 |
| 6 | Proline | 24.37 ± 1.54 | 24.27 ± 0.98 (0.997) | 18.8 ± 0.16 (<0.001) | <0.001 | 1.00 | 0.77 |
| 7 | Pyroglutamic acid | 109.7 ± 3.58 | 109.6 ± 0.16 (1.000) | 110.7 ± 1.65 (0.180) | 0.174 | 0.89 | 0.64 |
| 8 | 4-Hydroxyproline | 0.08 ± 0.03 | 0.10 ± 0.01 (0.858) | 0.09 ± 0.01 (<0.001) | <0.001 | 1.25 | 1.06 |
| 9 | Serine | 0.90 ± 0.12 | 1.02 ± 0.10 (0.999) | 1.40 ± 0.24 (<0.001) | <0.001 | 1.14 | 1.56 |
| 10 | Threonine | 14.51 ± 1.25 | 17.15 ± 0.95 (0.948) | 18.80 ± 1.22 (0.040) | 0.059 | 1.18 | 1.29 |
| 11 | r-Aminobutric acid | 0.10 ± 0.03 | 0.10 ± 0.02 (1.000) | 0..06 ± 0.03 (0.001) | 0.001 | 1.09 | 1.06 |
| 12 | Phenylalanine | 11.32 ± 0.72 | 10.75 ± 0.93 (0.607) | 11.12 ± 0.50 (0.002) | 0.001 | 0.97 | 1.19 |
| 13 | Cysteine | 6.24 ± 0.39 | 6.25 ± 0.74 (1.000) | 4.85 ± 0.56 (0.007) | 0.007 | 1.00 | 0.78 |
| 14 | Aspartic acid | 25.01 ± 1.36 | 24.99 ± 0.71 (1.000) | 21.7 ± 1.10 (<0.001) | <0.001 | 1.00 | 0.87 |
| 15 | Glutamic acid | 81.23 ± 4.11 | 83.28 ± 4.46 (0.990) | 75.2 ± 2.40 (0.002) | 0.002 | 1.03 | 0.93 |
| 16 | Asparagine | 87.84 ± 9.97 | 100.7 ± 8.36 (0.679) | 77.59 ± 5.84 (0.026) | 0.074 | 1.94 | 2.33 |
| 17 | Glutamine | 2.46 ± 0.57 | 2.65 ± 0.58 (0.998) | 4.24 ± 2.59 (0.001) | 0.002 | 1.51 | 2.22 |
| 18 | Lysine | 51.85 ± 1.78 | 51.18 ± 3.23 (0.996) | 50.76 ± 2.11 (0.002) | 0.001 | 1.39 | 2.56 |
| 19 | Tryptophane | 10.57 ± 0.29 | 10.12 ± 1.58 (0.983) | 9.59 ± 0.57 (0.033) | 0.027 | 0.96 | 0.91 |

^a^Values as each analyte concentration (μg/ml). ^b^One way ANOVA comparing the mean values of control group and treated group with MNPs@SiO_2_(RITC) of 10 μg/ml. ^c^One way ANOVA comparing the mean values of control group and treated group with MNPs@SiO_2_(RITC) of 100 μg/ml. ^d^One way ANOVA comparing the mean values of group with MNPs@SiO_2_(RITC) of 10 μg/ml and treated group with MNPs@SiO_2_(RITC) of 100 μg/ml. ^e^Ratio values of analyte in treated groups with MNPs@SiO_2_(RITC) to corresponding mean values in the control group.
