# Supplementary figures and images for: Silica-coated magnetic nanoparticles activate microglia and induce neurotoxic d-serine secretion
Source: Part Fibre Toxicol. 2021 Aug 12;18:30. doi: 10.1186/s12989-021-00420-3 (PMC8359100; doi:10.1186/s12989-021-00420-3)

**a**

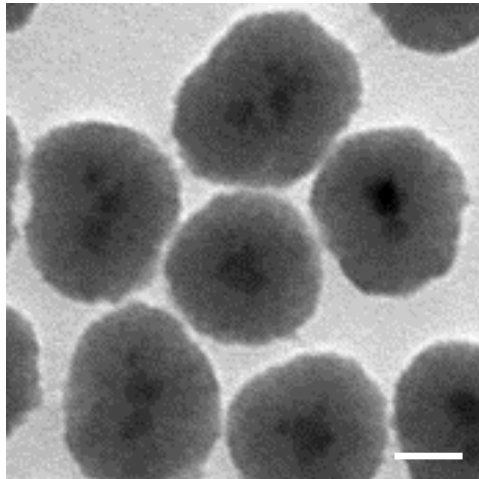

**b**

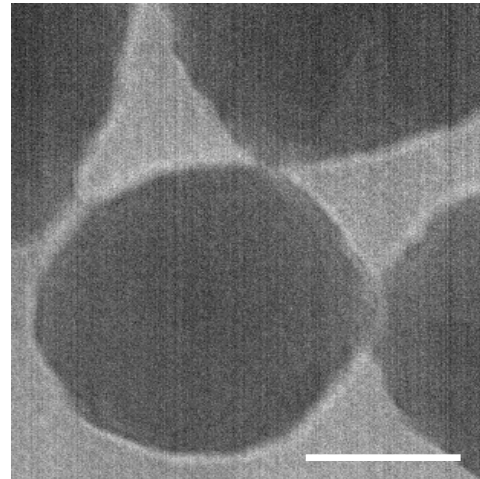

Supplement: Supplementary file 7 — Additional file 7: Supplementary Figure 1. Transmission electron microscope images for a﻿ MNPs@SiO2(RITC) and ﻿b SiO2 NPs. Scale bar = 20 nm. [file 12989_2021_420_MOESM7_ESM.pdf]

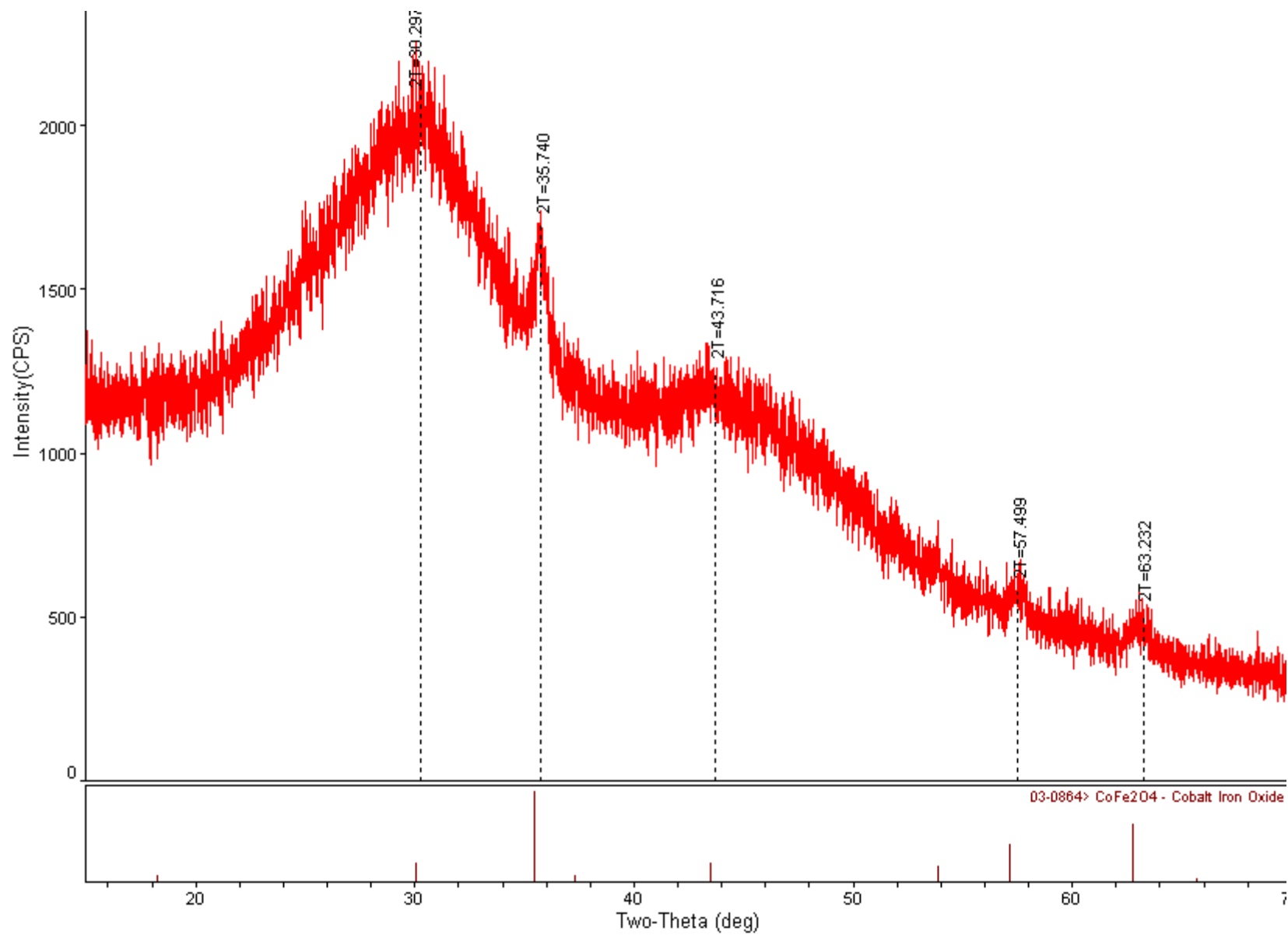

Supplement: Supplementary file 8 — Additional file 8: Supplementary Figure 2. XRD spectrum of MNPs@SiO2(RITC). Peaks in 30o, 36o, 44o, 57o and 64 o are specific patterns of cobalt ferrite and the broad peak between 20o and 40o indicate the amorphous silica beads. [file 12989_2021_420_MOESM8_ESM.pdf]

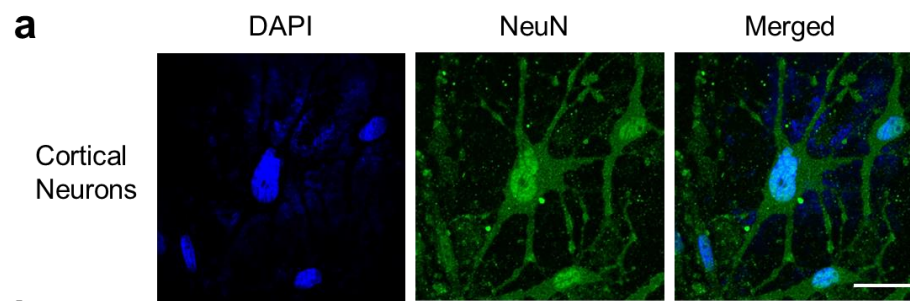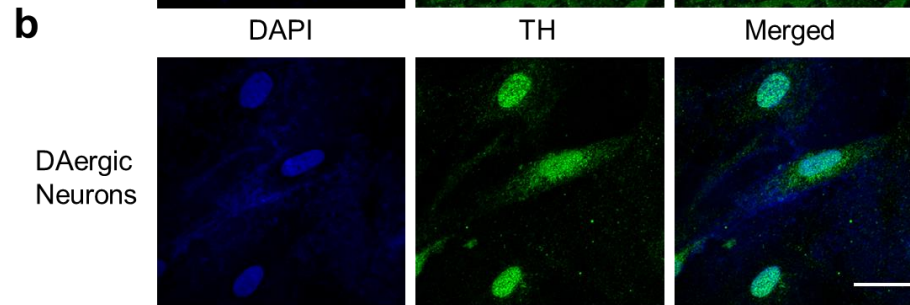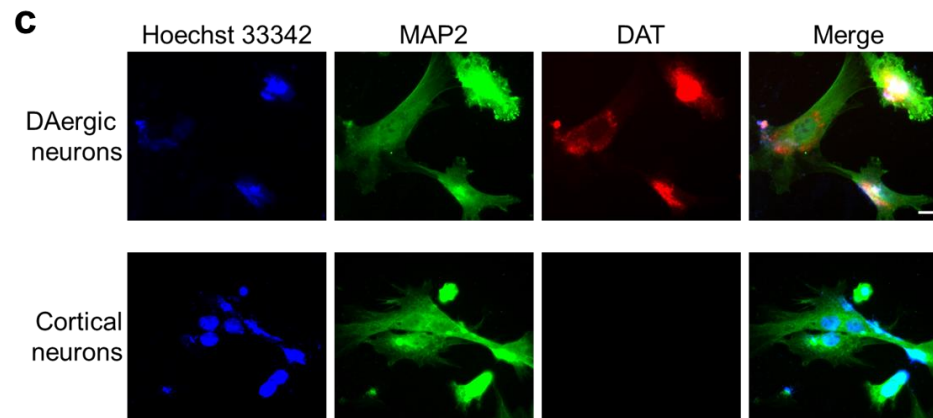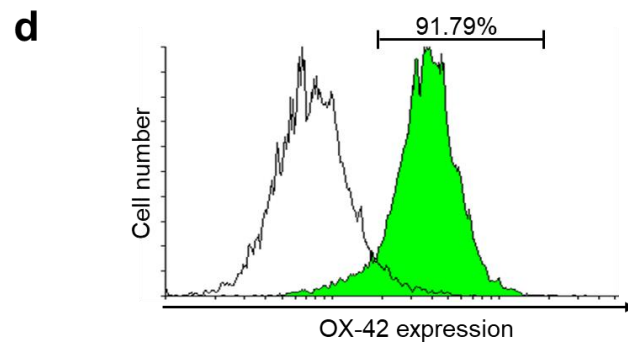

Supplement: Supplementary file 9 — Additional file 9: Supplementary Figure 3. Characterization of primary rat neuronal cells and microglia. a﻿ Immunofluorescence of cortical neurons stained with anti-NeuN antibody. b﻿ Immunofluorescence of DAergic neurons stained with anti-TH antibody. Scale bar = 10 μm. c﻿ Immunofluorescence of cortical and DAergic neurons stained with anti-MAP2 and DAT antibodies. Scale bar = 20 μm. d﻿ FACS analysis for OX-42 expression in primary rat microglia. Percentage indicates positively stained population of cells. [file 12989_2021_420_MOESM9_ESM.pdf]

**a**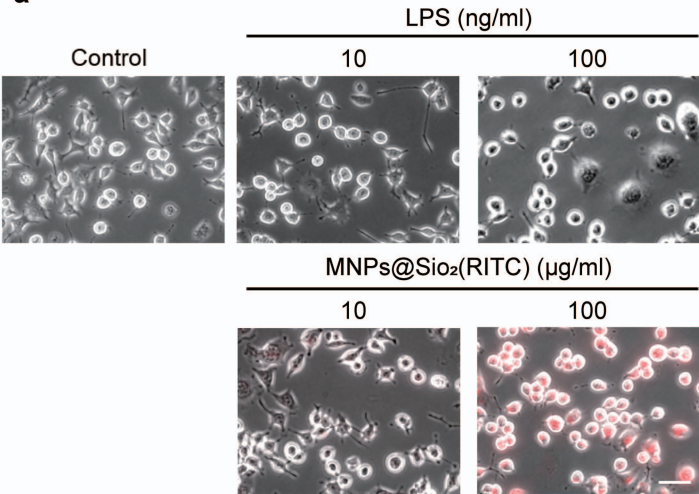**b**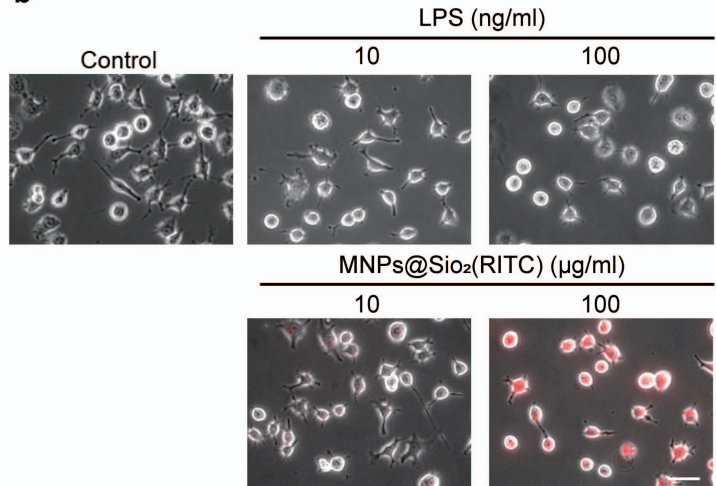

Supplement: Supplementary file 10 — Additional file 10: Supplementary Figure 4. Microglia activation after MNPs@SiO2(RITC) treatment. Morphological analysis of a﻿ BV2 and b﻿ primary rat microglia. Scale bar = 50 μm. Red; MNPs@SiO2(RITC). [file 12989_2021_420_MOESM10_ESM.pdf]

Silica NPs ( $\mu\text{g/ml}$ )

0

10

100

BV2

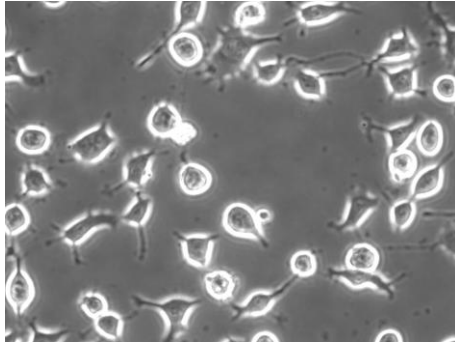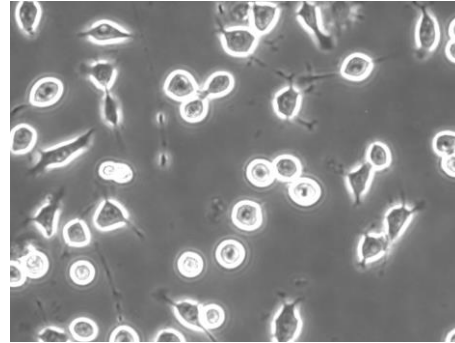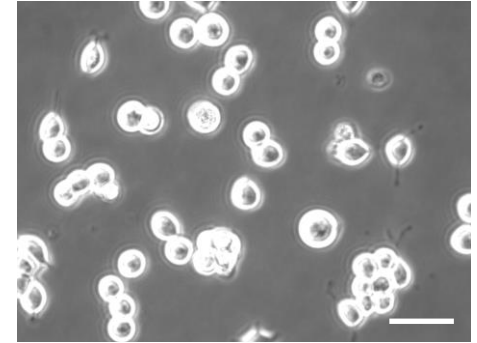

Primary  
rat microglia

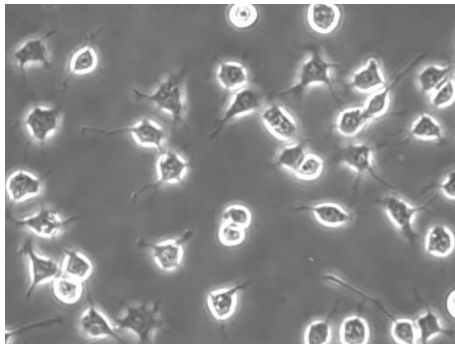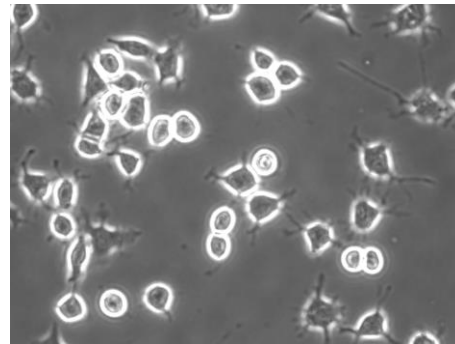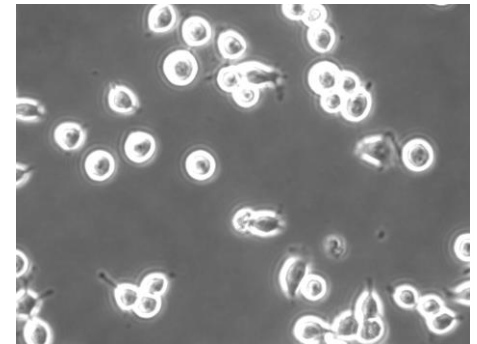

Supplement: Supplementary file 11 — Additional file 11: Supplementary Figure 5. Microglia activation after SiO2 NPs treatment. Morphological analysis of BV2 and primary rat microglia. Scale bar = 50 μm. [file 12989_2021_420_MOESM11_ESM.pdf]

**a**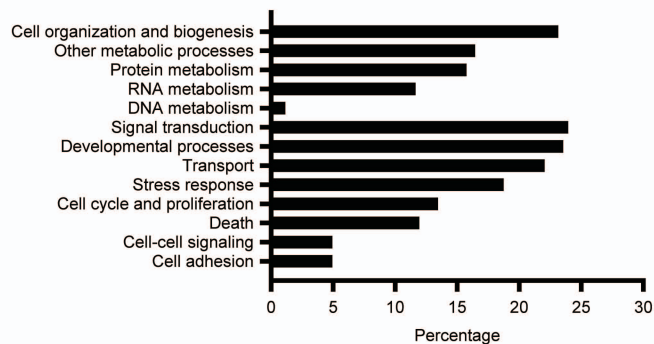**c**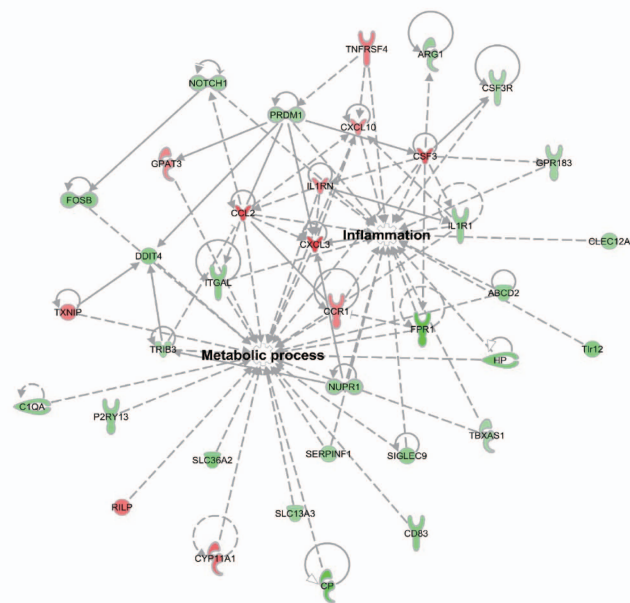**b**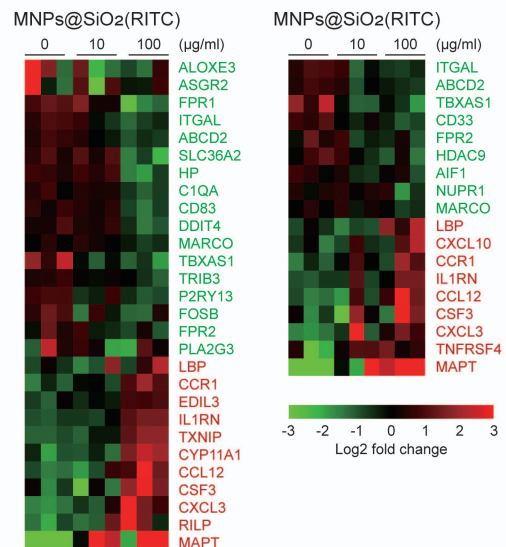**d**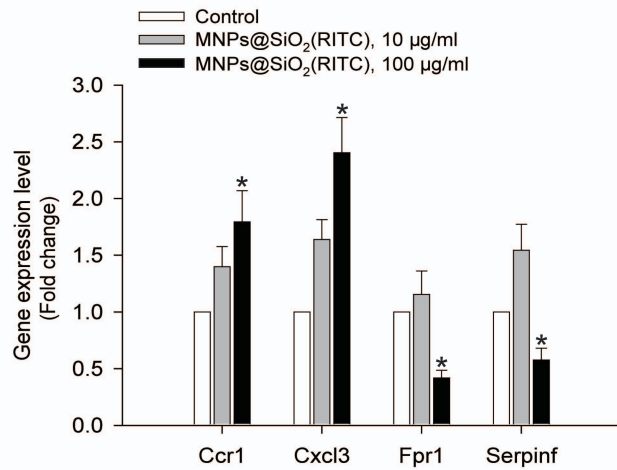

Supplement: Supplementary file 12 — Additional file 12: Supplementary Figure 6. RNA-seq-based transcriptome analysis of BV2 cells treated with MNPs@SiO2(RITC) for 12 h. ﻿a Categorization of DEGs identified by RNA-seq. DEGs were analyzed using the MGI GO tool. Metabolism-related genes are highlighted. b﻿ Heat map of DEGs (fold change > 1.5 or <–1.5), including 29 DEGs related to metabolism (left panel) and 18 DEGs related to inflammation (right panel), in control cells and 100 and 10 µg/ml MNPs@SiO2(RITC)-treated cells. ﻿c Metabolism- and inflammation-related gene network for 100 µg/ml-treated BV2 cells constructed by IPA. The red and green areas indicate the up- and downregulated genes, respectively. [file 12989_2021_420_MOESM12_ESM.pdf]

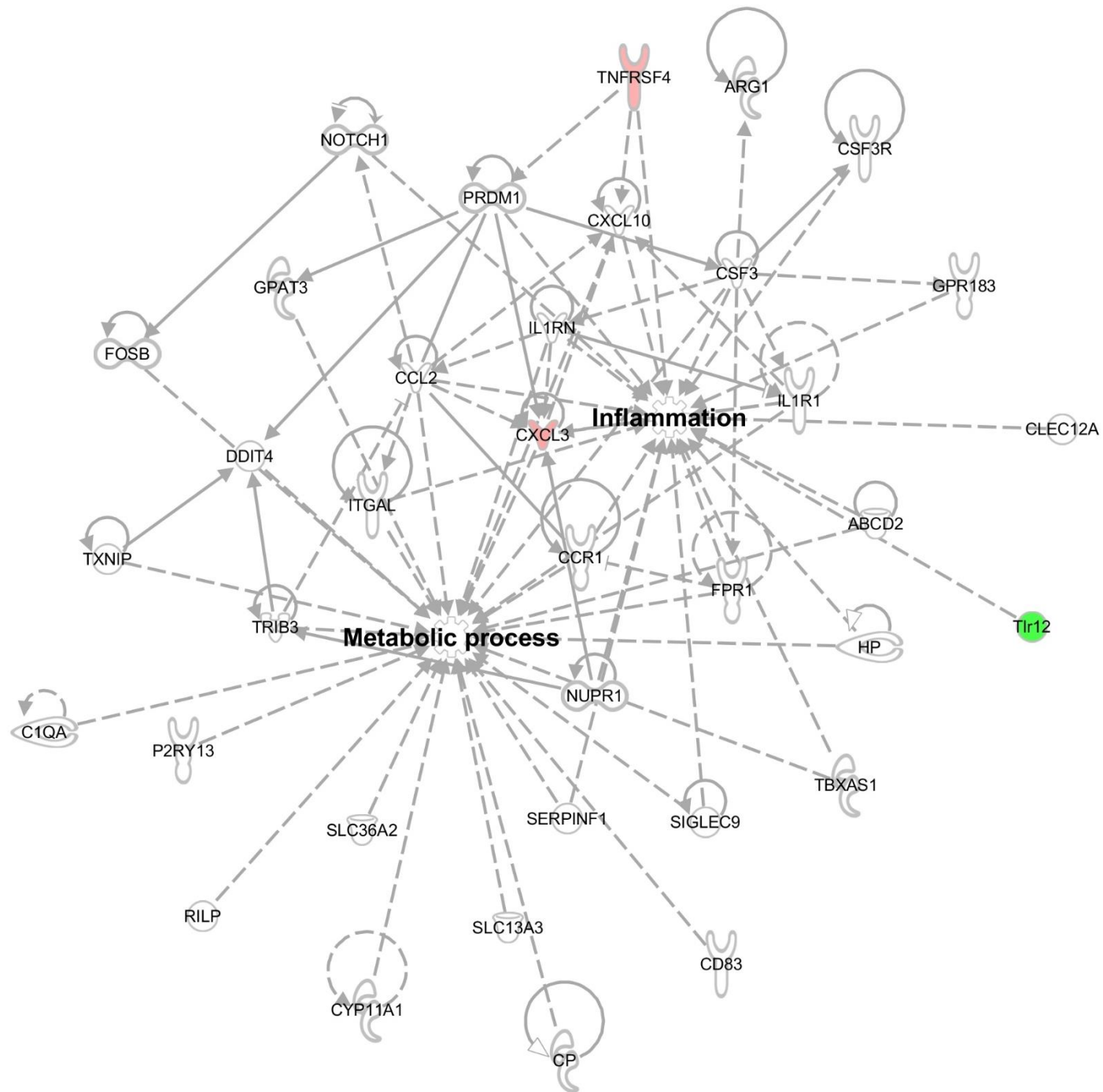

Supplement: Supplementary file 13 — Additional file 13: Supplementary Figure 7. Transcriptomic network of 10 µg/ml treated BV2 cells. Metabolism and inflammation related genes and network of 10 µg/ml treated BV2 cells was constructed algorithmically by IPA. Red and green areas indicate up- and downregulated genes, respectively. [file 12989_2021_420_MOESM13_ESM.pdf]

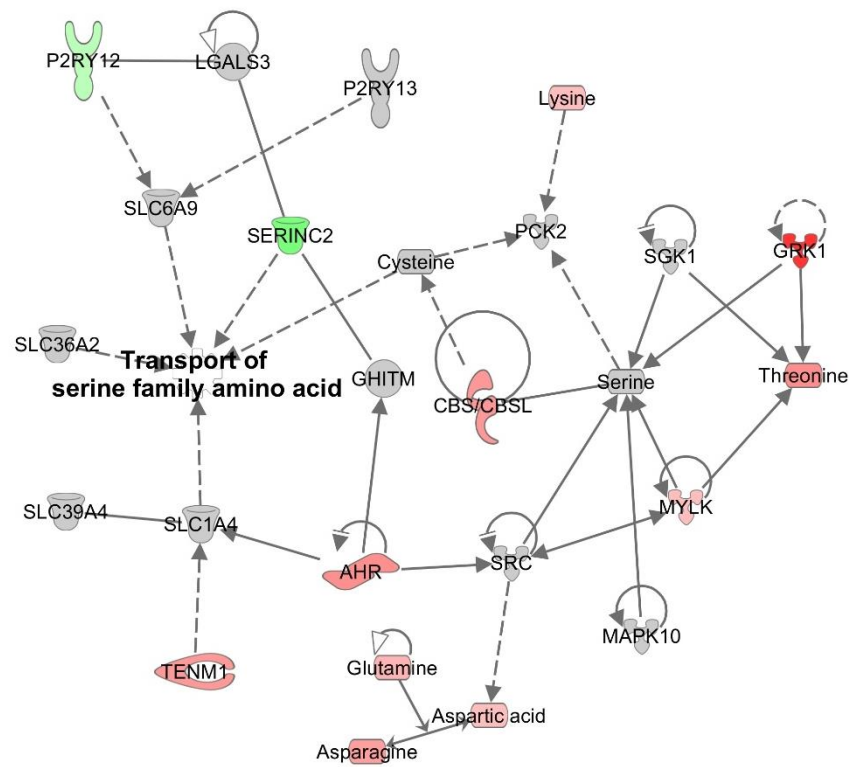

Supplement: Supplementary file 14 — Additional file 14: Supplementary Figure 8. Combinatorial analysis of RNA-seq and intracellular amino acid profiles 10 µg/ml MNPs@SiO2(RITC) treated BV2. Transcriptome combined with amino acids profiles network were constructed algorithmically by IPA in 10 µg/ml MNPs@SiO2(RITC) treated BV2. Red and green areas indicate up- and downregulated genes, respectively. Cut off fold change ± 1.5 for genes and ± 1.2 for amino acids were used. [file 12989_2021_420_MOESM14_ESM.pdf]

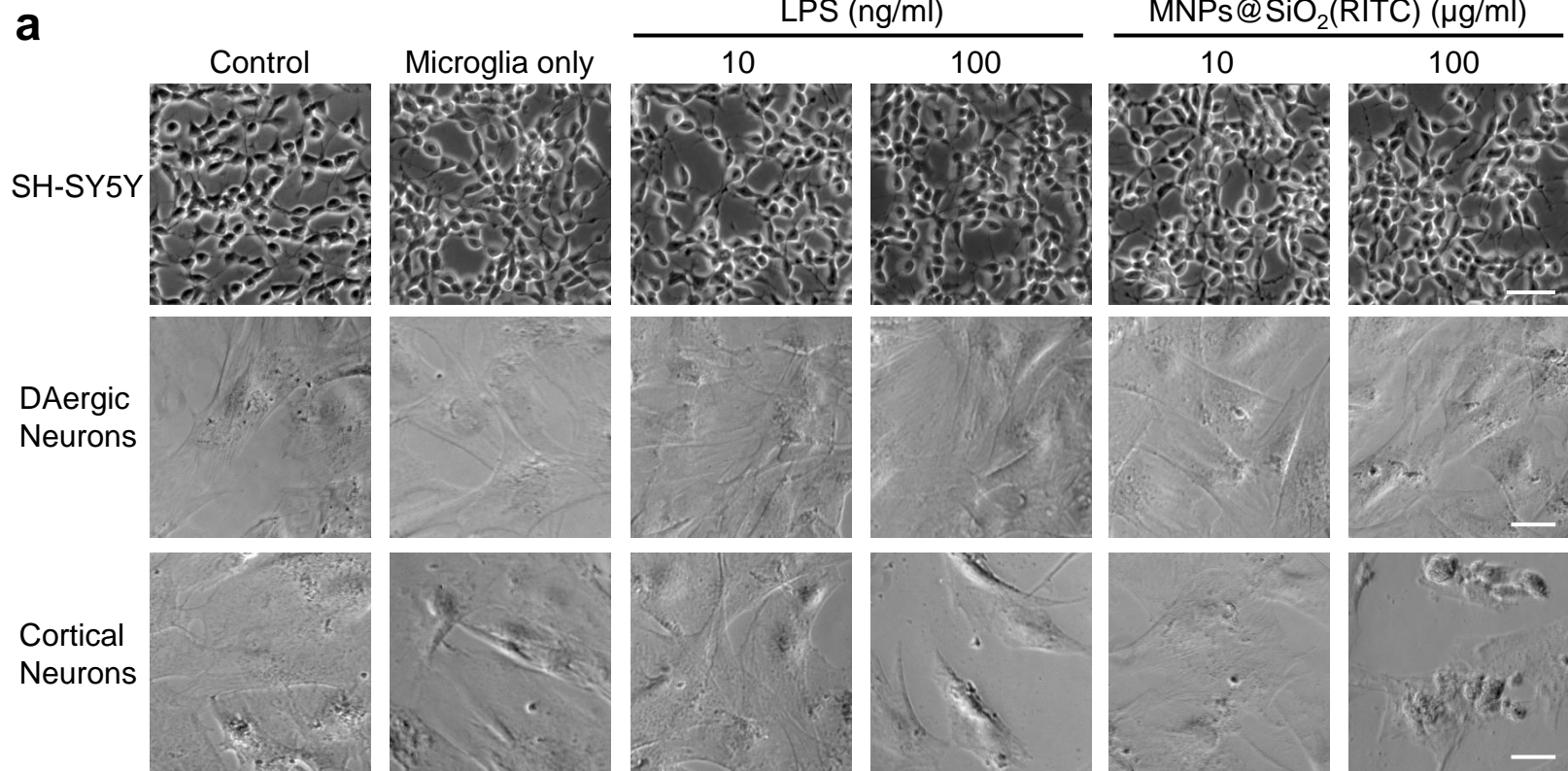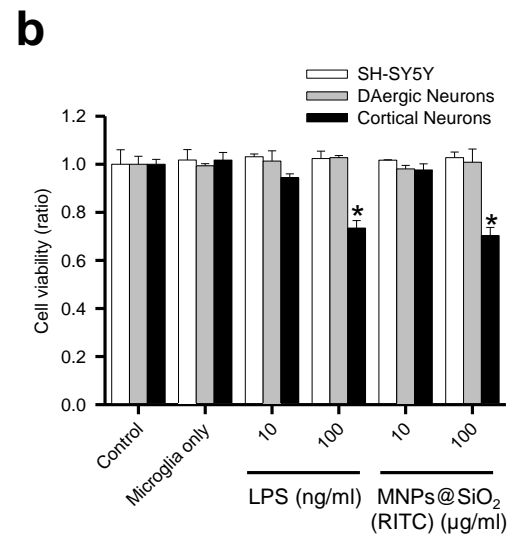

Supplement: Supplementary file 15 — Additional file 15: Supplementary Figure 9. Evaluation of cytotoxicity on neuronal cells in coculture system with activated microglia. ﻿a Morphological analysis and cell density observation of SH-SY5Y, DAergic, and cortical neurons. Scale bar = 50 μm. ﻿b MTS assay for evaluation of cocultured neurons viability. Data were normalized with non-treated control and represent mean ± SD of three independent experiments. *p < 0.05 vs. non-treated control. [file 12989_2021_420_MOESM15_ESM.pdf]

# MNPs@SiO<sub>2</sub>(RITC) (μg/ml) treated microglia co-culture

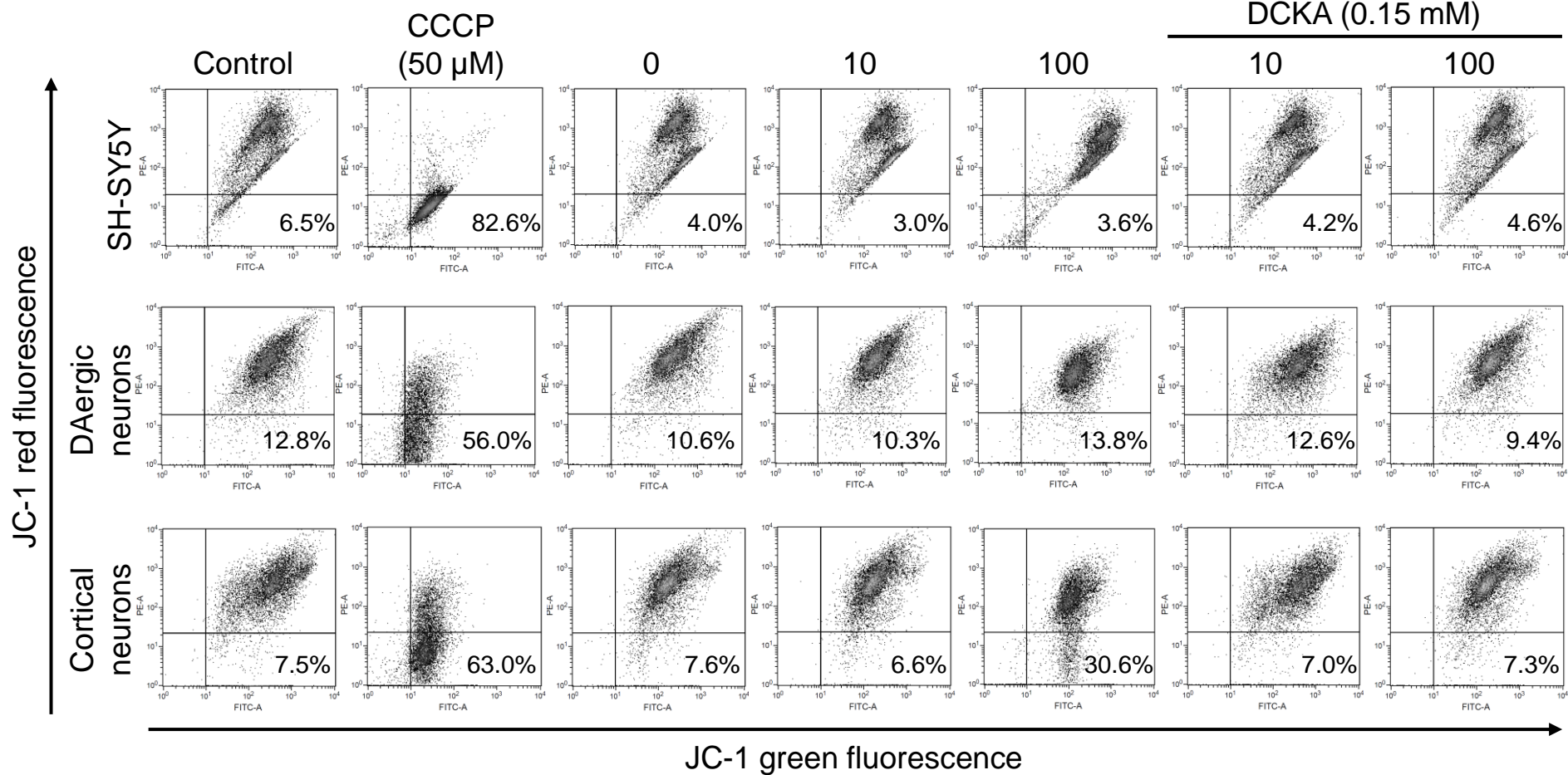

Supplement: Supplementary file 16 — Additional file 16: Supplementary Figure 10. Evaluation of changes in mitochondria membrane potential in MNPs@SiO2(RITC) treated primary rat microglia cocultured neuronal cells. Mitochondria membrane potentials were analyzed using 5,5’,6,6’-tetrachloro-1,1’,3,3’- tetraethylbenzimidazolyl carbocyanine iodide (JC-1). Red fluorescence (high membrane potential) and green fluorescence (low membrane potential) were measured by FACS. Percentage indicates the portion of cells with low mitochondria membrane potential. Carbonyl cyanide m-chlorophenyl hydrazine (CCCP) were used as positive control for decreasing mitochondria membrane potential. [file 12989_2021_420_MOESM16_ESM.pdf]

**a**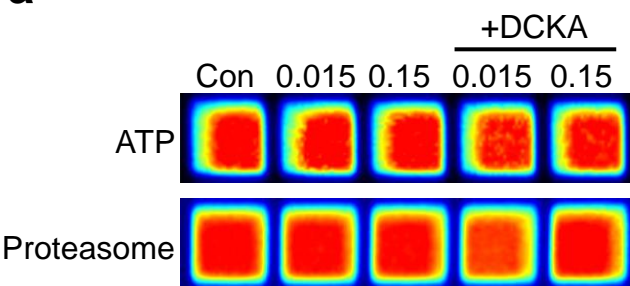**b**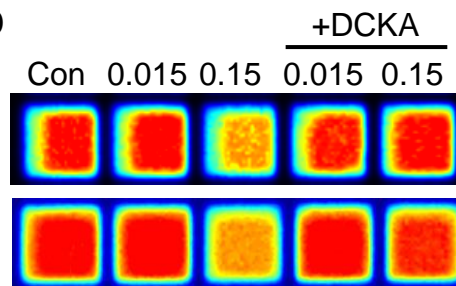**c**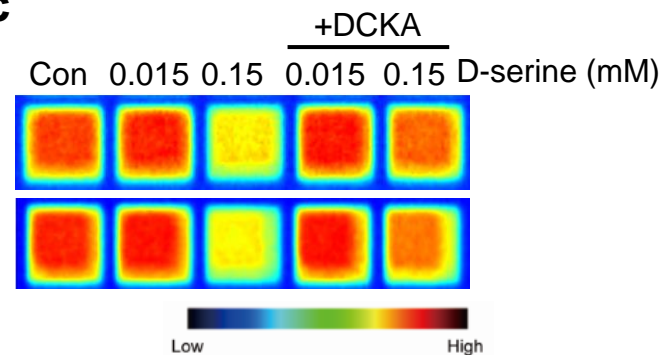**d**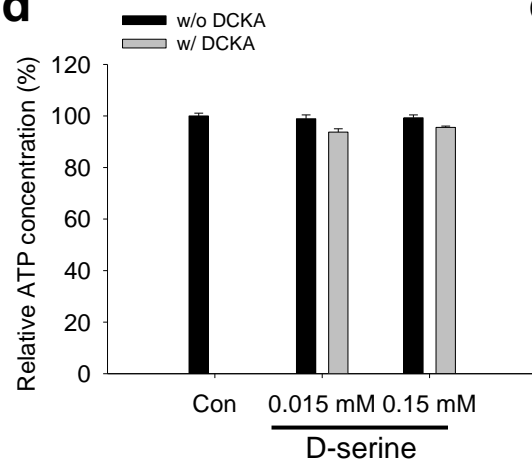**e**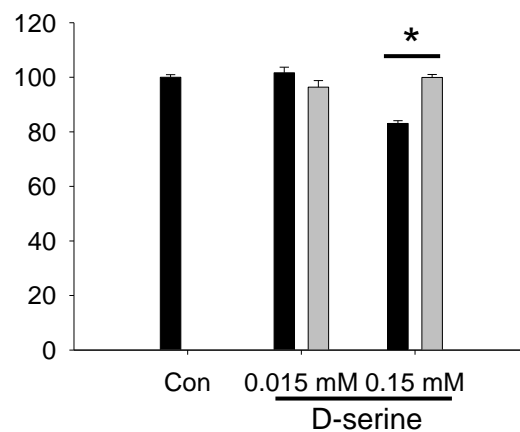**f**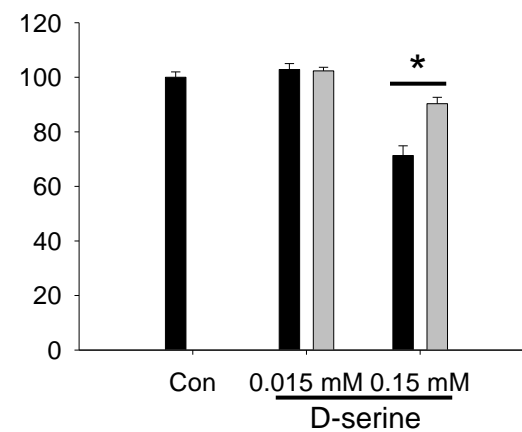**g**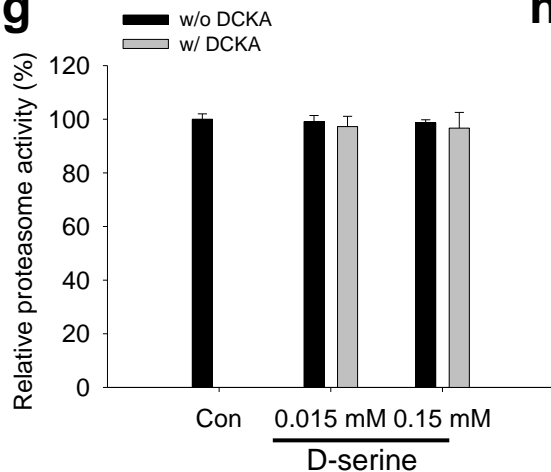**h**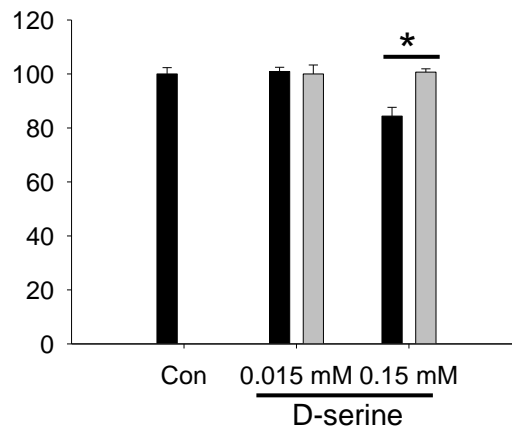**i**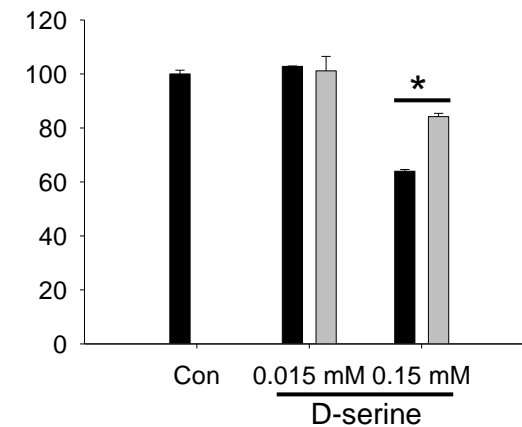

Supplement: Supplementary file 17 — Additional file 17: Supplementary Figure 11. Evaluation of the intracellular ATP level and proteasomal activity in D-serine treated neuronal cells. Luminescence images of the ATP level and proteasomal activity for ﻿a SH-SY5Y, b﻿ DAergic neurons, and c﻿ cortical neurons. Statistical analysis of ATP levels in ﻿d SH-SY5Y, e﻿ DAergic, and f cortical neurons. Statistical analysis of proteasomal activity in g SH-SY5Y, h DAergic, and i cortical neurons. *p < 0.05 vs. without DCKA. Con: neuronal cells only. The experiments were repeated three times independently, and a representative result is shown. [file 12989_2021_420_MOESM17_ESM.pdf]

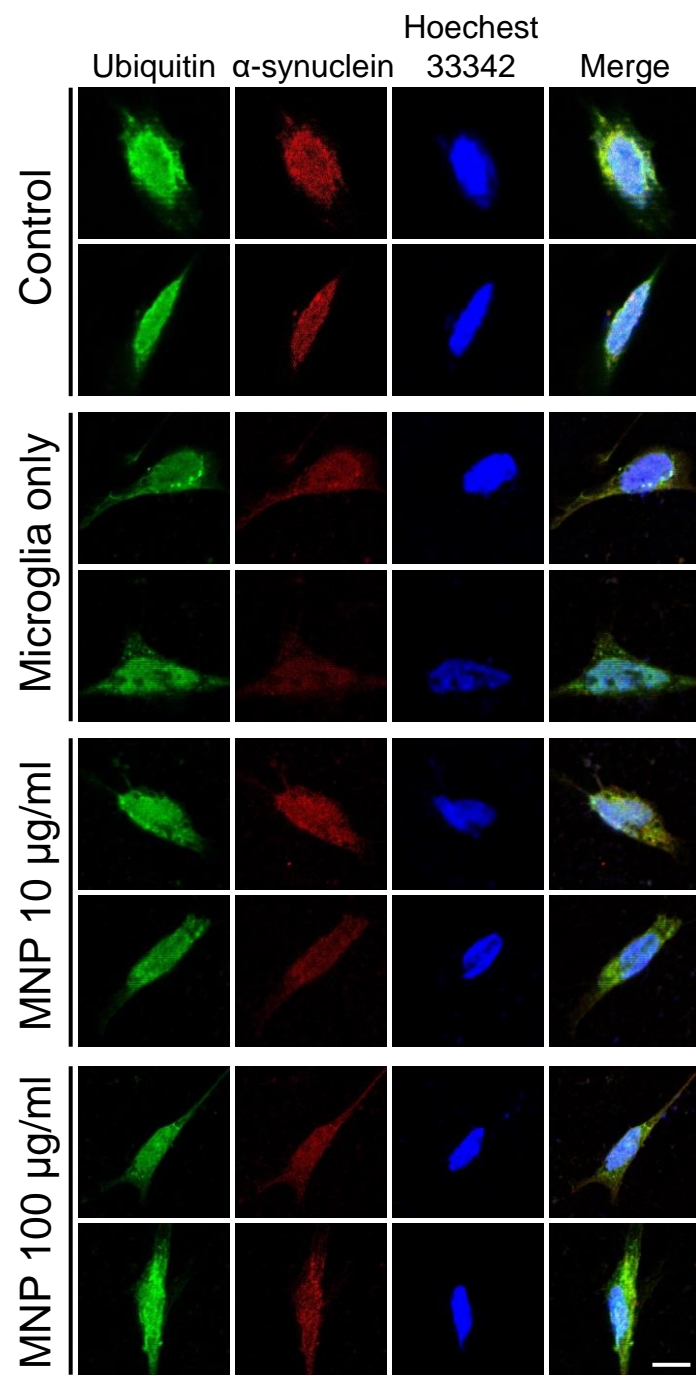

Supplement: Supplementary file 18 — Additional file 18: Supplementary Figure 12. Immunocytochemistry analysis in MNPs@SiO2(RITC) treated microglia cocultured SH-SY5Y cells. Images were acquired by immunostaining with ubiquitin (green), α-synuclein (red), and nucleus (blue) Scale bar = 10 μm. [file 12989_2021_420_MOESM18_ESM.pdf]

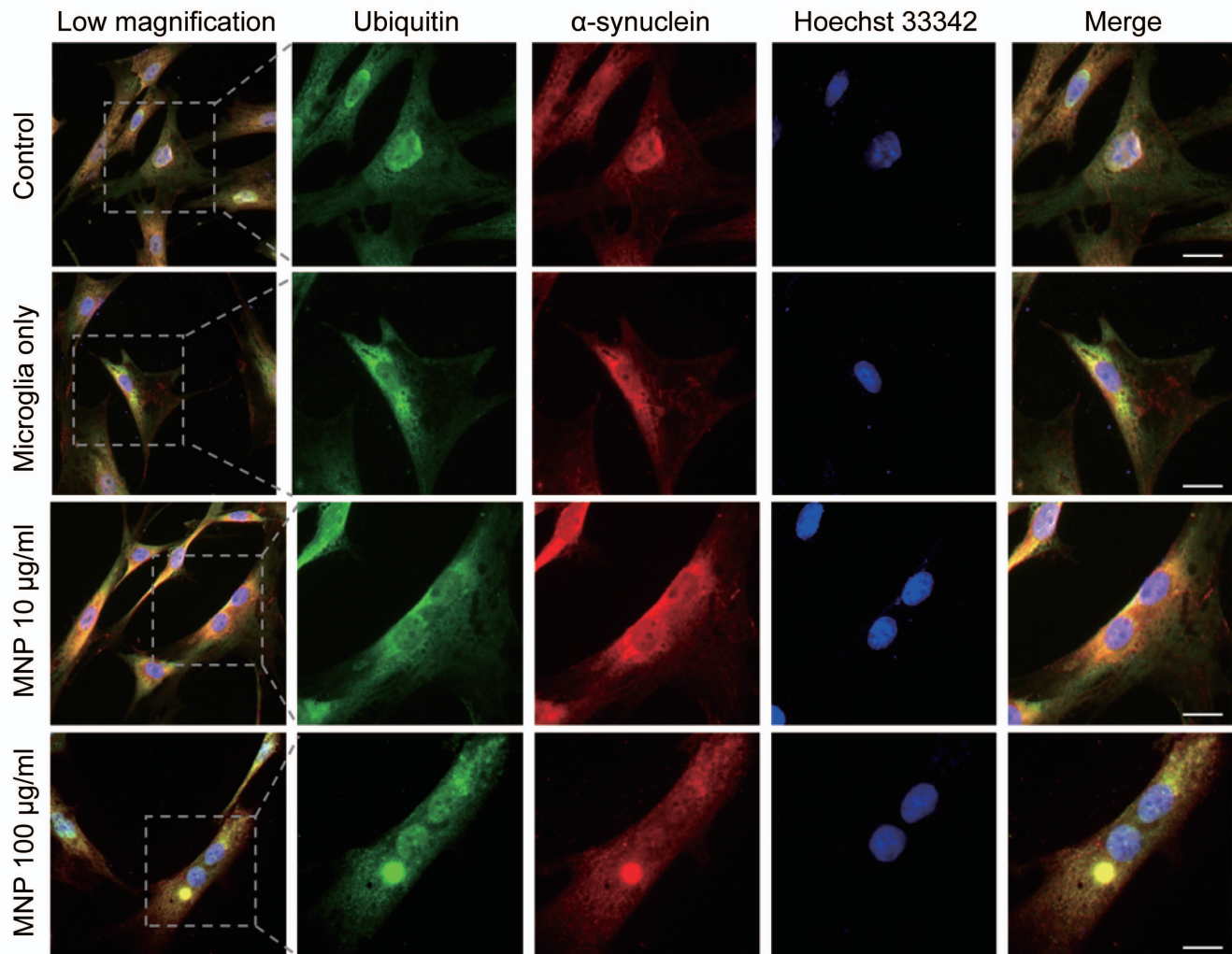

Supplement: Supplementary file 19 — Additional file 19: Supplementary Figure 13. Inclusion body formation in MNPs@SiO2(RITC) treated microglia cocultured DAergic neurons. Images of inclusion body formation were acquired by immunostaining with ubiquitin (green), α-synuclein (red), and nucleus (blue) Scale bar = 10 μm. [file 12989_2021_420_MOESM19_ESM.pdf]

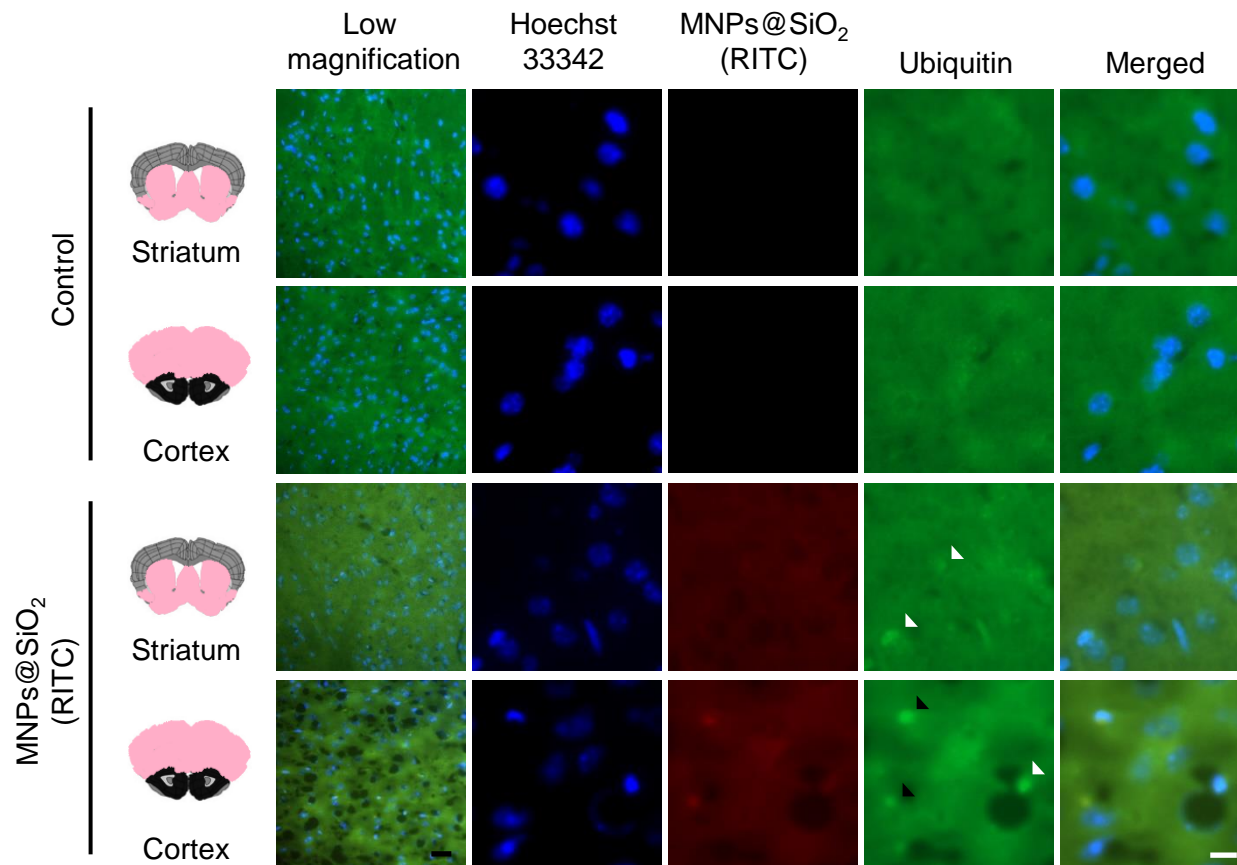

Supplement: Supplementary file 20 — Additional file 20: Supplementary Figure 14. Inclusion body formation in MNPs@SiO2(RITC) treated mice brains. Images of inclusion body formation were acquired by immunostaining with ubiquitin (green), MNPs@SiO2(RITC) (red), and nucleus (blue). Black scale bar = 100 μm. White scale bar = 10 μm. White arrowhead indicates inclusion body without colocalized to MNPs@SiO2(RITC) and black arrowhead indicates inclusion body with colocalized to MNPs@SiO2(RITC). [file 12989_2021_420_MOESM20_ESM.pdf]

**a**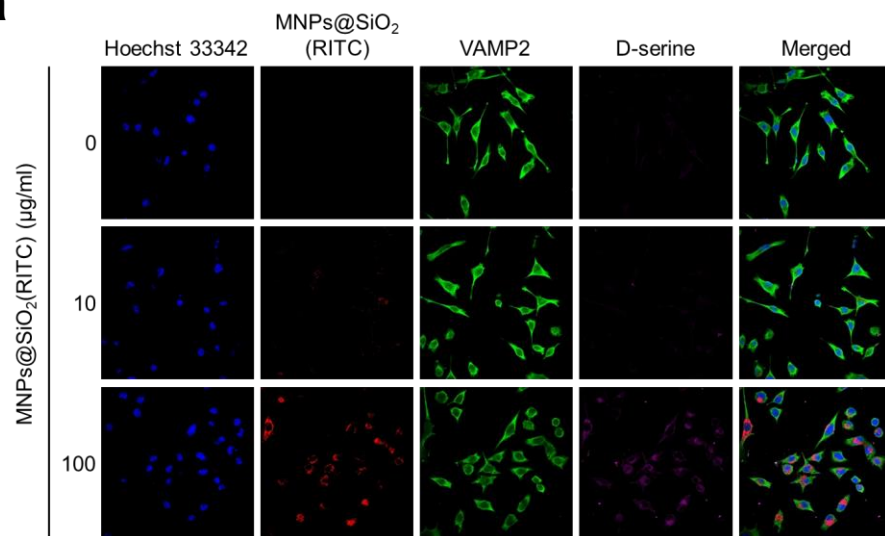**b**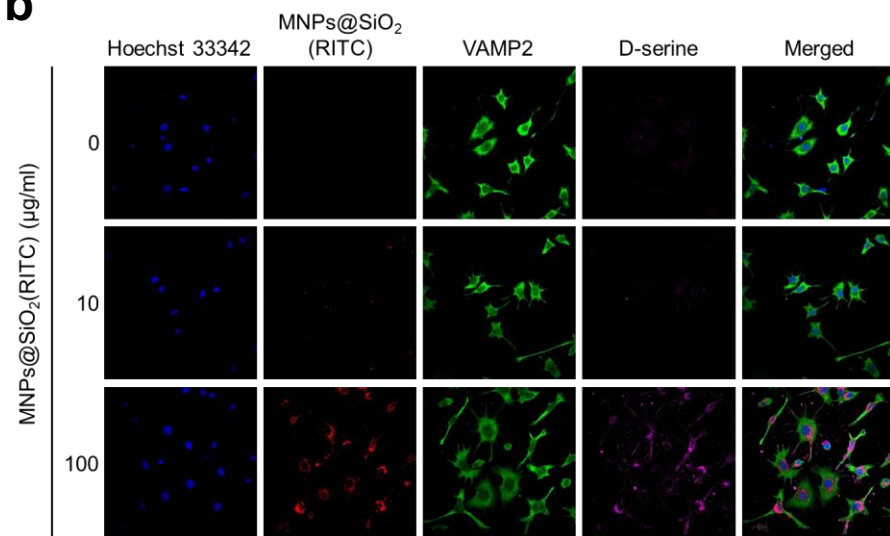**c**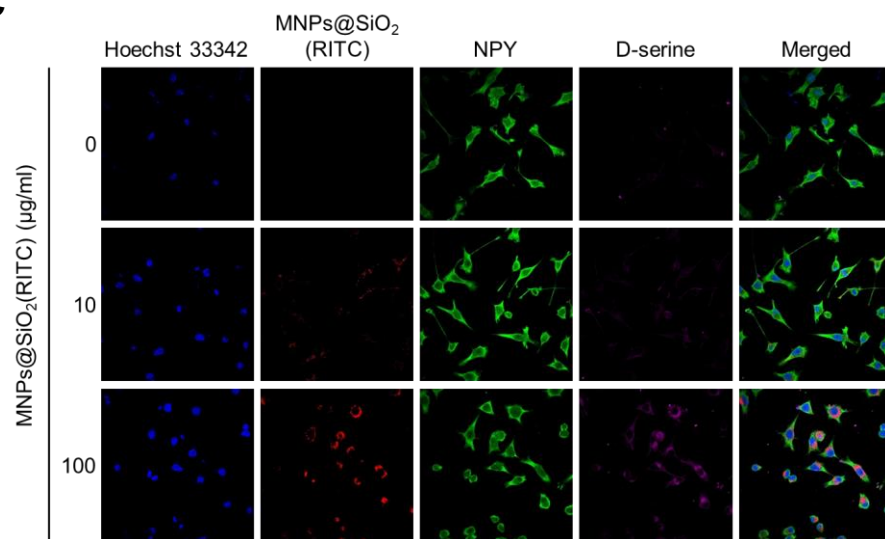**d**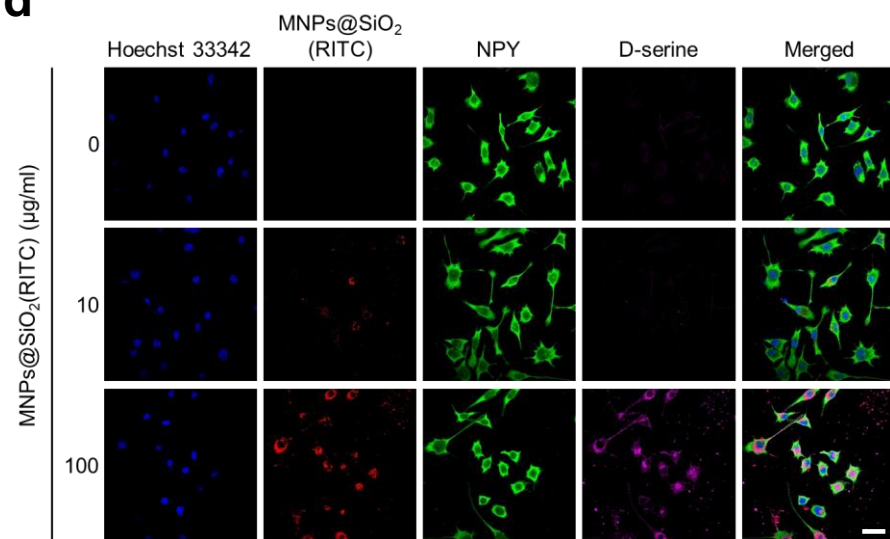

Supplement: Supplementary file 21 — Additional file 21: Supplementary Figure 15. Immunostaining analysis for exocytic vesicle marker and D-serine﻿ in MNPs@SiO2(RITC) treated microglia. Images of exocytic vesicle marker and D-serine﻿ were acquired by immunostaining with MNPs@SiO2(RITC) (red), VAMP2 or NPY (green), D-serine﻿ (violet), and nucleus (blue). Stained with VAMP2 in ﻿a BV2 and ﻿b primary rat microglia and stained with NPY in ﻿c BV2 and d﻿ primary rat microglia were analysed. Scale bar = 20 μm. [file 12989_2021_420_MOESM21_ESM.pdf]
